# Author Correction: A practical guide for mutational signature analysis in hematological malignancies

**DOI:** 10.1038/s41467-019-11468-3

**Published:** 2019-07-25

**Authors:** Francesco Maura, Andrea Degasperi, Ferran Nadeu, Daniel Leongamornlert, Helen Davies, Luiza Moore, Romina Royo, Bachisio Ziccheddu, Xose S. Puente, Herve Avet-Loiseau, Peter J. Campbell, Serena Nik-Zainal, Elias Campo, Nikhil Munshi, Niccolò Bolli

**Affiliations:** 1 Myeloma Service, Department of Medicine, Memorial Sloan Kettering Cancer Center, New York, 10065 NY USA; 2 Department of Oncology and Hemato-Oncology, University of Milan, Via Festa del Perdono 7, Milan, 20122 Italy; 3 Cancer, Ageing, and Somatic Mutation Programme, Wellcome Sanger Institute, Hinxton, Cambridgeshire CB10 1SA UK; 4 Department of Medical Genetics, Cambridge University Hospitals NHS Foundation Trust, Cambridge, CB2 0QQ UK; 5 MRC Cancer Unit, University of Cambridge, Hutchison/MRC Research Centre, Cambridge Biomedical Campus, Cambridge, CB2 0XZ UK; 6 Patologia Molecular de Neoplàsies Limfoides, Institut d’Investigacions Biomèdiques August Pi i Sunyer (IDIBAPS), 08036 Barcelona, Spain; 7 Centro de Investigación Biomédica en Red de Cáncer (CIBERONC), 28029 Madrid, Spain; 8 Barcelona Supercomputing Center (BSC), Joint BSC-CRG-IRB Research Program in Computational Biology, 08036 Barcelona, Spain; 9 Department of Clinical Oncology and Hematology, Fondazione IRCCS Istituto Nazionale dei Tumori, Milan, 20133 Italy; 10 Unitat Hematopatologia, Hospital Clínic of Barcelona, Universitat de Barcelona, 08036 Barcelona, Spain; 11 Departamento de Bioquimica y Biologia Molecular, Instituto Universitario de Oncologia (IUOPA), Universidad de Oviedo, Oviedo, 33003 Spain; 12 IUC-Oncopole, and CRCT INSERM U1037, 31100 Toulouse, France; 13 Jerome Lipper Multiple Myeloma Center, Dana–Farber Cancer Institute, Harvard Medical School, Boston, 02215 MA USA; 14 Veterans Administration Boston Healthcare System, West Roxbury, 02130 MA USA

**Keywords:** Cancer genomics, Haematological cancer, Leukaemia, Myeloma, Genomics

Correction to: *Nature Communications* 10.1038/s41467-019-11037-8, published online 5 July 2019.

The original version of this Article contained an error in the spelling of the author Peter J Campbell, which was incorrectly given as Peter J Cambell. This has now been corrected in both the PDF and HTML versions of the Article.

