# Supplementary material for: A practical guide for mutational signature analysis in hematological malignancies
Source: Nat Commun. 2019 Jul 5;10:2969. doi: 10.1038/s41467-019-11037-8 (PMC6611883; doi:10.1038/s41467-019-11037-8)
Supplement: Supplementary file 1 — Supplementary information [file 41467_2019_11037_MOESM1_ESM.pdf]

Maura et al

“A practical guide for mutational signature analysis in hematological malignancies”

Supplementary Information

Supplementary Table 1. Summary of clinical and biological features of 30 multiple myeloma samples.

Sample ID	Age	Sex	Phase	Ig Type	Isotype	Cytogenetic
**PD26400a**	69	M	SMM	IgG	Kappa	HRD*
**PD26401a**	74	M	SMM	IgG	Kappa	
**PD26402a**	55	F	SMM	IgG	Kappa	t(11;14)
**PD26403a**	57	F	SMM	IgG	Kappa	HRD
**PD26404a**	64	M	SMM	IgG	Kappa	HRD
**PD26405a**	64	F	SMM	IgA	Kappa	t(4;14)
**PD26406a**	61	M	SMM	IgG	Kappa	t(4;14)
**PD26407a**	49	M	SMM	IgG	Kappa	HRD
**PD26408a**	53	M	SMM	IgG	Kappa	HRD
**PD26409a**	64	F	MGUS	IgG	Kappa	HRD
**PD26410d**	66	M	RR	IgG	Lambda	HRD
**PD26411c**	64	M	RR	IgA	Lambda	HRD
**PD26412a**	50	M	RR	IgG	Kappa	HRD
**PD26414a**	54	F	RR	IgA	Kappa	t(4;14)
**PD26415c**	55	M	RR	IgG	Kappa	HRD
**PD26416d**	52	F	RR	IgG	Kappa	HRD
**PD26418a**	71	M	RR	IgA	Kappa	t(11;14)
**PD26419a**	65	F	DG	LC	Kappa	t(8q24;14)
**PD26420a**	56	M	RR	IgM	Kappa	t(11;14)
**PD26422d**	70	M	RR	IgA	Kappa	t(11;14)
**PD26423e**	54	M	RR	IgG	Kappa	HRD
**PD26424a**	63	M	SMM	IgG	Kappa	HRD
**PD26425e**	63	M	RR	IgA	Kappa	t(11;14)
**PD26426e**	77	M	RR	IgG	Kappa	HRD
**PD26427a**	51	F	DG	LC	Lambda	t(11;14)
**PD26428a**	41	M	DG	IgG	Kappa	t(11;14)
**PD26429a**	64	F	DG	IgG	Kappa	HRD
**PD26432c**	61	M	RR	IgA	Lambda	HRD
**PD26434c**	58	M	DG	IgG	Kappa	t(11;14)
**PD26435c**	65	M	RR	IgA	Kappa	HRD

*HRD= hyperdiploid

Supplementary Figure 1. Mutational signature matrix decomposition. Schematic diagram explaining the mutational signature matrix decomposition according to the formula $C \approx SE$, where C is the catalogue matrix, with mutation types as rows and samples as columns, S is the signature matrix, with mutation types as rows and signatures as columns, and E is the exposure matrix, with signatures as rows and samples as columns.

$$C \approx SE$$

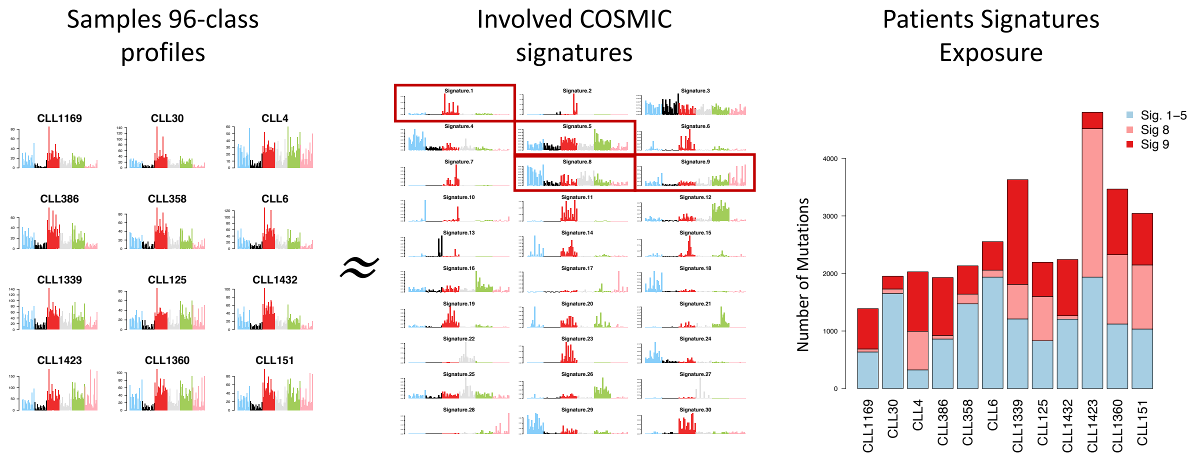

Supplementary Figure 2. Recurrent flat signatures in hematological cancers. The 96-mutational profile of Signature 3, 5 and 8, all considered as “flat” signatures due to the relatively even contribution of each trinucleotide context (<0.05).

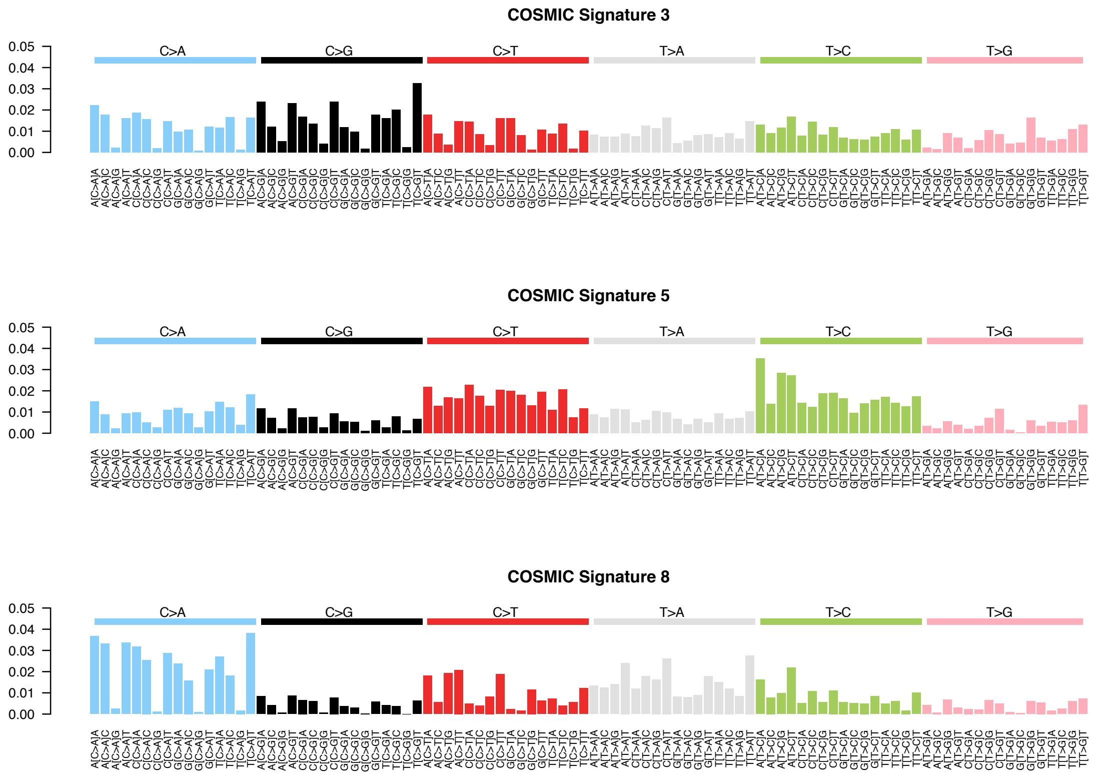

Supplementary Figure 3. Reconstruction error using MM mutational signatures. Reconstruction error of the 30 MM samples computed as KL divergence (KLD, top row), root mean squared error (RMSE, middle row) and cosine similarity (Cos Sim, bottom row), and using different combinations of the flat mutational signatures reported across different hematological malignancies (COSMIC Signatures 3, 5 and 8): including signature 3, 5 and 8 (with Sig. 3, 5 and 8), including only signatures 3 and 5 (without Sig. 8), including only signatures 5 and 8 (without Sig. 3), and including only signatures 3 and 8 (without Sig. 5). The first column (a,b,c) shows the reconstruction error in the three settings as boxplots, with p-values computed using a two-sided paired t-test; here, boxes show median, 1st and 3rd quartiles and whiskers are placed at the most external data point within 1.5*(75th-25th percentile). The second column (d,e,f) compares the reconstruction error in the setting "with Sig. 3 and 8" against "without Sig. 8". The third column (g,h,i) compares "with Sig. 3, 5 and 8" against "without Sig. 3"; and the fourth column (j,k,l) compares "with Sig. 3, 5 and 8" against "without Sig. 5". Black lines indicate the line $x = y$. The data point corresponding to sample PD26419a is marked with a red dot.

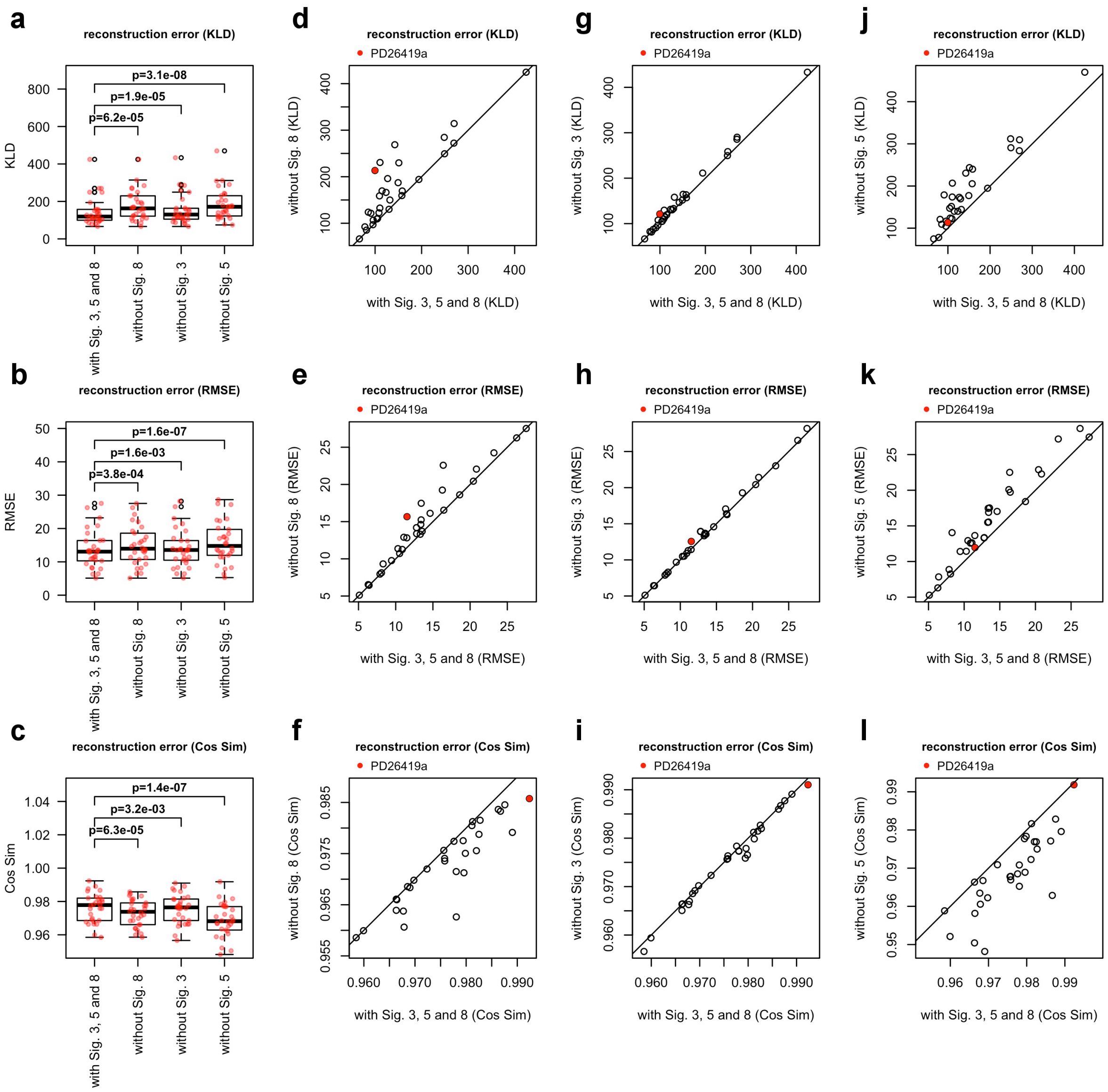
a
reconstruction error (KLD)
p=3.1e-08
p=1.9e-05
p=6.2e-05
KLD
with Sig. 3, 5 and 8
without Sig. 8
without Sig. 3
without Sig. 5
b
reconstruction error (RMSE)
p=1.6e-07
p=1.6e-03
p=3.8e-04
RMSE
c
reconstruction error (Cos Sim)
p=1.4e-07
p=3.2e-03
p=6.3e-05
Cos Sim
d
PD26419a
without Sig. 8 (KLD)
with Sig. 3, 5 and 8 (KLD)
e
without Sig. 8 (RMSE)
with Sig. 3, 5 and 8 (RMSE)
f
without Sig. 8 (Cos Sim)
with Sig. 3, 5 and 8 (Cos Sim)
g
without Sig. 3 (KLD)
h
without Sig. 3 (RMSE)
i
without Sig. 3 (Cos Sim)
j
without Sig. 5 (KLD)
k
without Sig. 5 (RMSE)
l
without Sig. 5 (Cos Sim)

Supplementary Figure 4. Mutational signature assignment in MM. Mutations assigned to signatures in the 30 MM samples, using different combinations of the mutational signatures 3 and 8: including only signature 8 (without Sig. 3), including both signatures 3 and 8 (with Sig. 3 and 8). Each dot is a sample. Black lines indicate the line $x = y$.

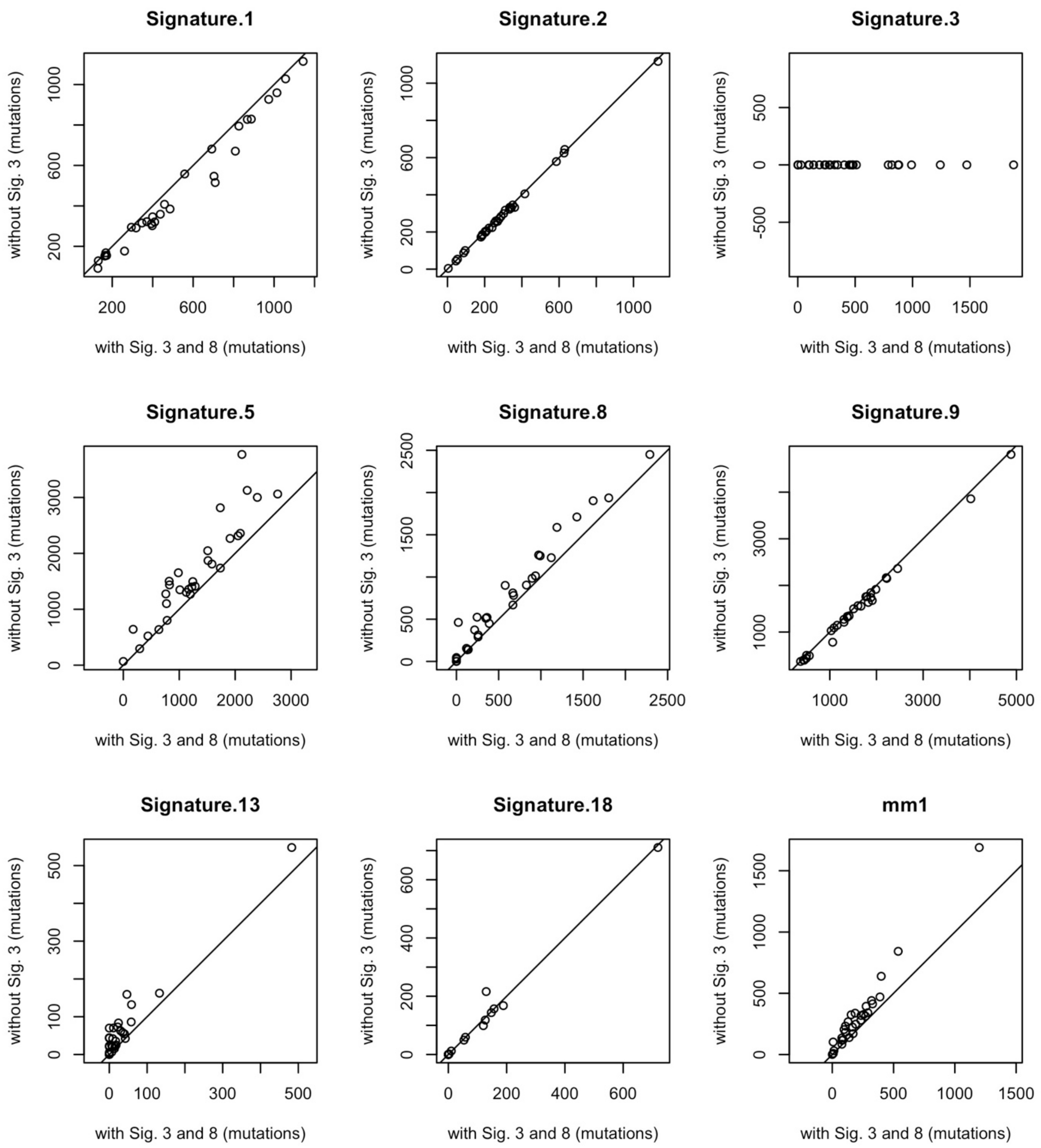

Supplementary Figure 5. Indel landscape in MM and BRCA-null breast cancer. We annotated the indel landscape according to the recently proposed classification [1] for all MMs (a), for PD26419a -the case that showed a uncertain HRD- (b) and for 2 BRCA-null breast cancers (c-d). In MMs and in PD26419a in particular we did not observe any enrichment for microhomology deletions as observed in the 2 BRCA1/BRCA2 deficient breast cancers.

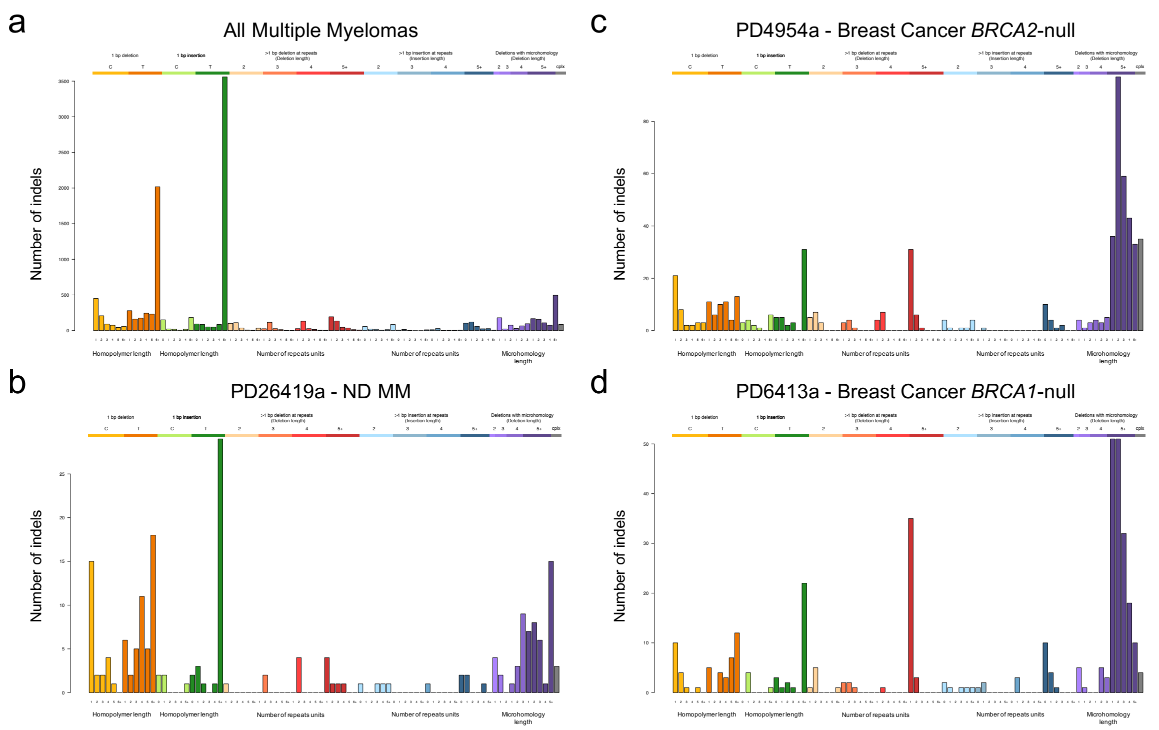

Supplementary Figure 6. The structural variant (SV) signatures for each MM case and for the 2 *BRCA*-null breast cancers. None of the MM samples had a SV signature profile similar to that observed among the *BRCA*-null breast cancers, that showed also a clear enrichment in term of SV prevalence.

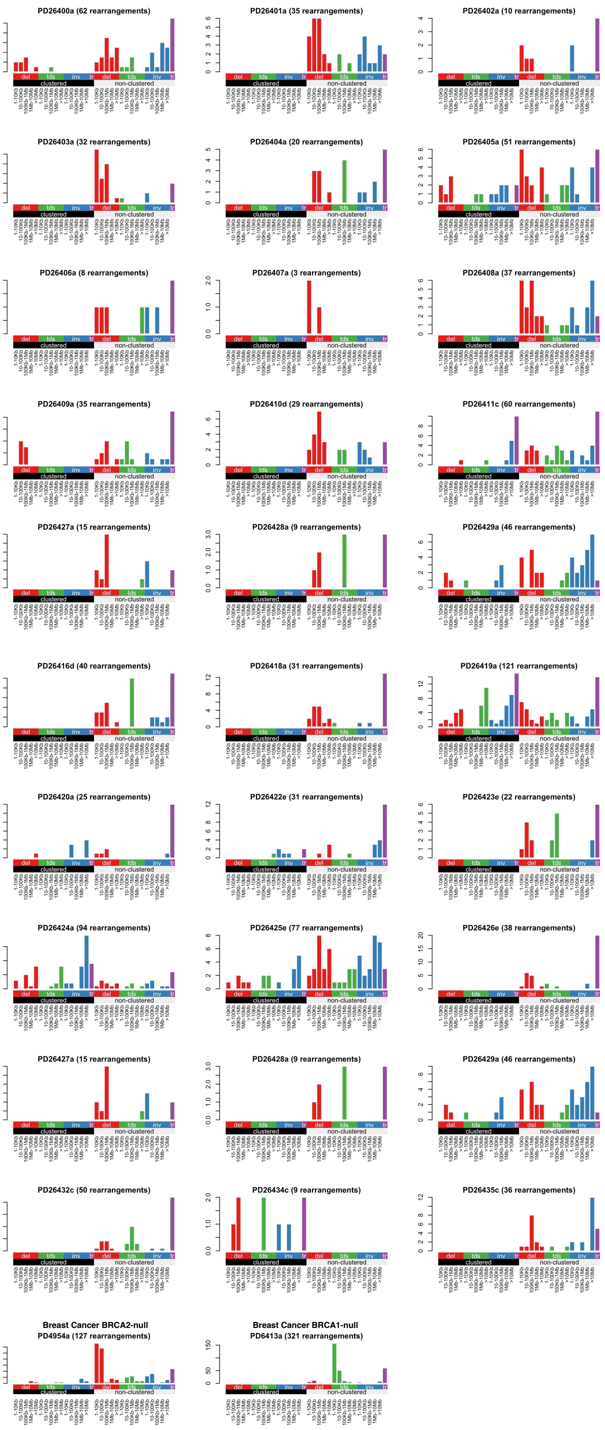

Supplementary Figure 7. AID mutational signatures. The 96-mutational profile of non-canonical AID (Signature 9, top) and canonical AID (bottom)[2,3]. The first is active all over the genome and represents one of the main mutational process in MM and M-CLL. Conversely, the second is active only as a localized mutational process within IGH/IGK/IGL regions and on few other off-target genes in cancer (i.e *BCL6*).

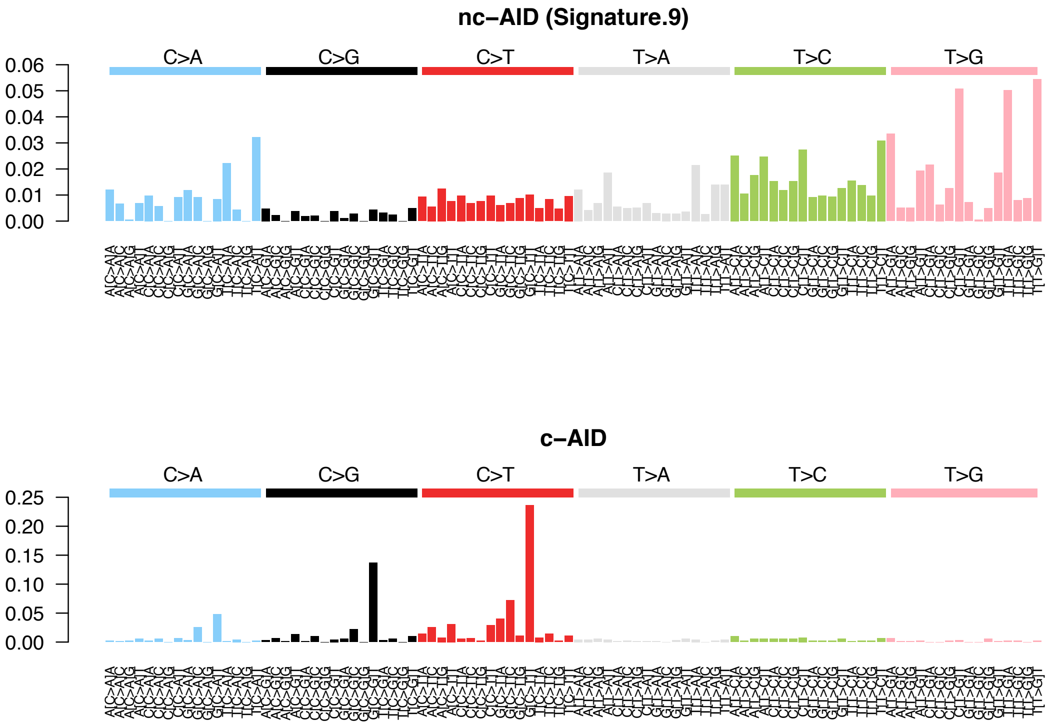

Supplementary Figure 8. CLL and MM mutation distribution on IGH. The SNV distribution on the IGH region of (a) M-CLLs (n=67), (b) U-CLLs (n=68) and (c) MMs (n=30). d) Relative and absolute mutation rate on coding gene within IGH locus. All cancers are subject to a different grade of c-AID activity, and, interestingly, in U-CLL cases this activity was mostly on the non-coding part compared to MM and M-CLL. e) The cancer cell fraction of all mutations within IGH/IGK/IGL loci. U-CLLs showed a significantly higher fraction of subclonal mutations compared to both M-CLL and MM (p<0.0001 and 0.01 respectively). P value were generated using the Wilcoxon test (R function wilcox.test)

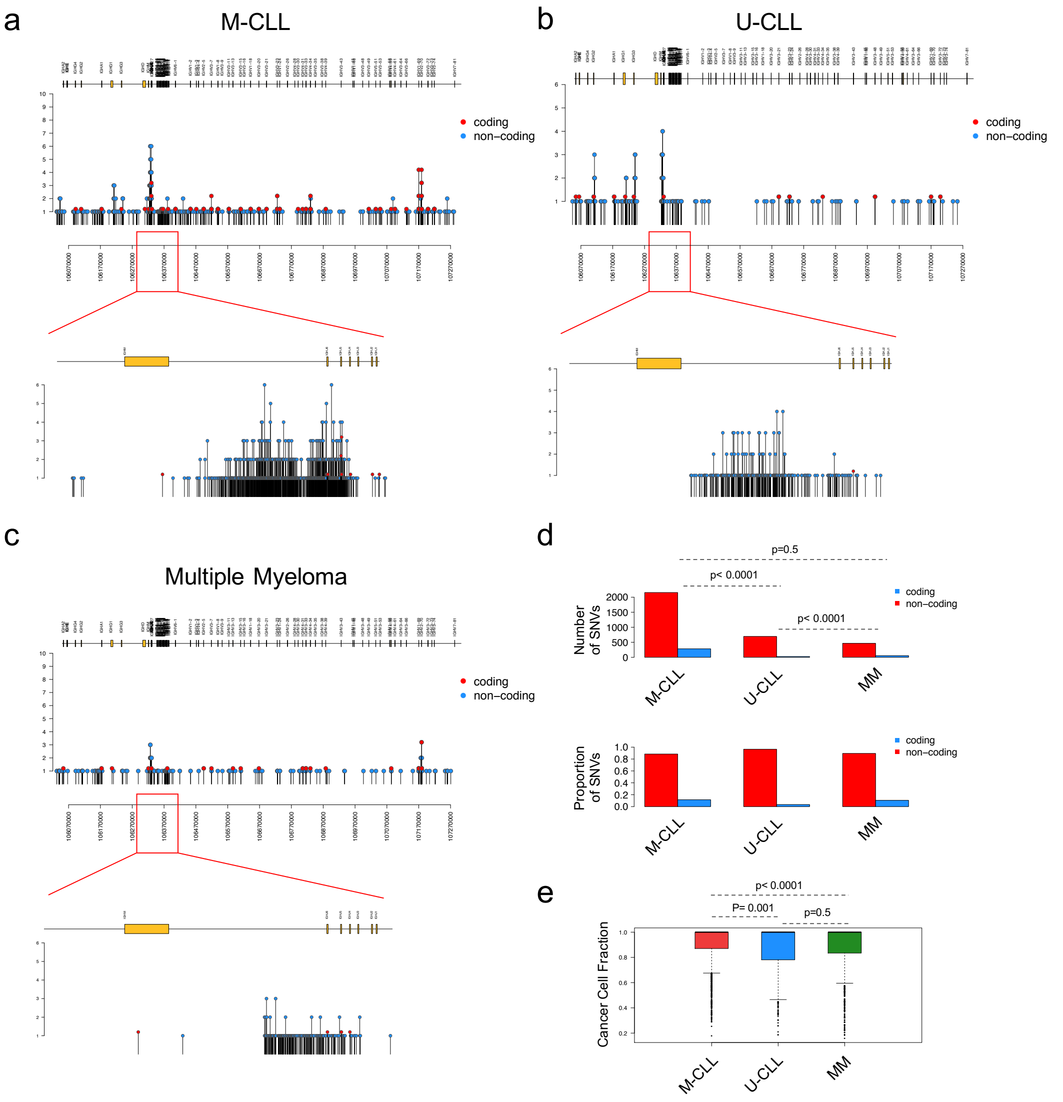

Supplementary Figure 9. Localized hypermutation on the IGH locus. In this cartoon, we summarized the VDJ recombination process. This recombination juxtaposes otherwise distant DNA segments, and once mapped to the germline reference genome the inter-mutational distance of c-AID localized hypermutation appears falsely high. The same rationale can be applied to other complex regions and events involving localized mutational events (i.e kataegis).

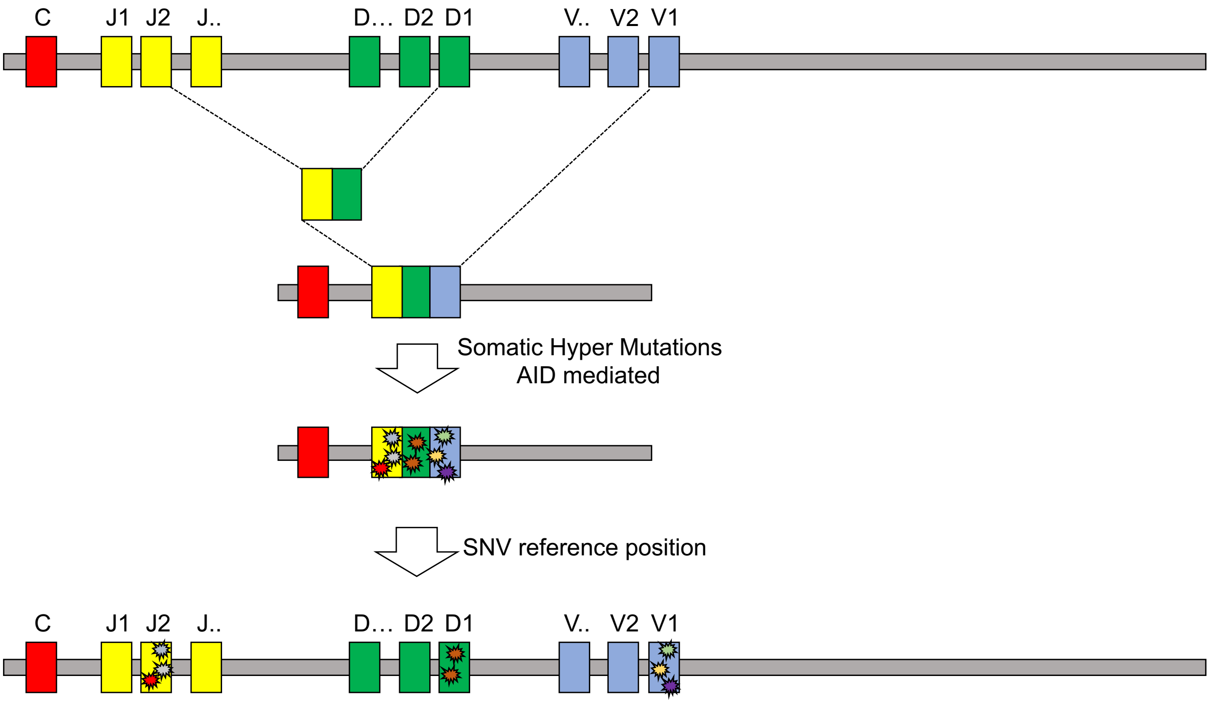

Supplementary Figure 10. BCL6 mutational distribution. a) distribution of SNVs within the *BCL6* gene locus for M-CLLs, U-CLLs and MMs. In contrast to the other entities, U-CLL did not show any evidence of localized hypermutation.

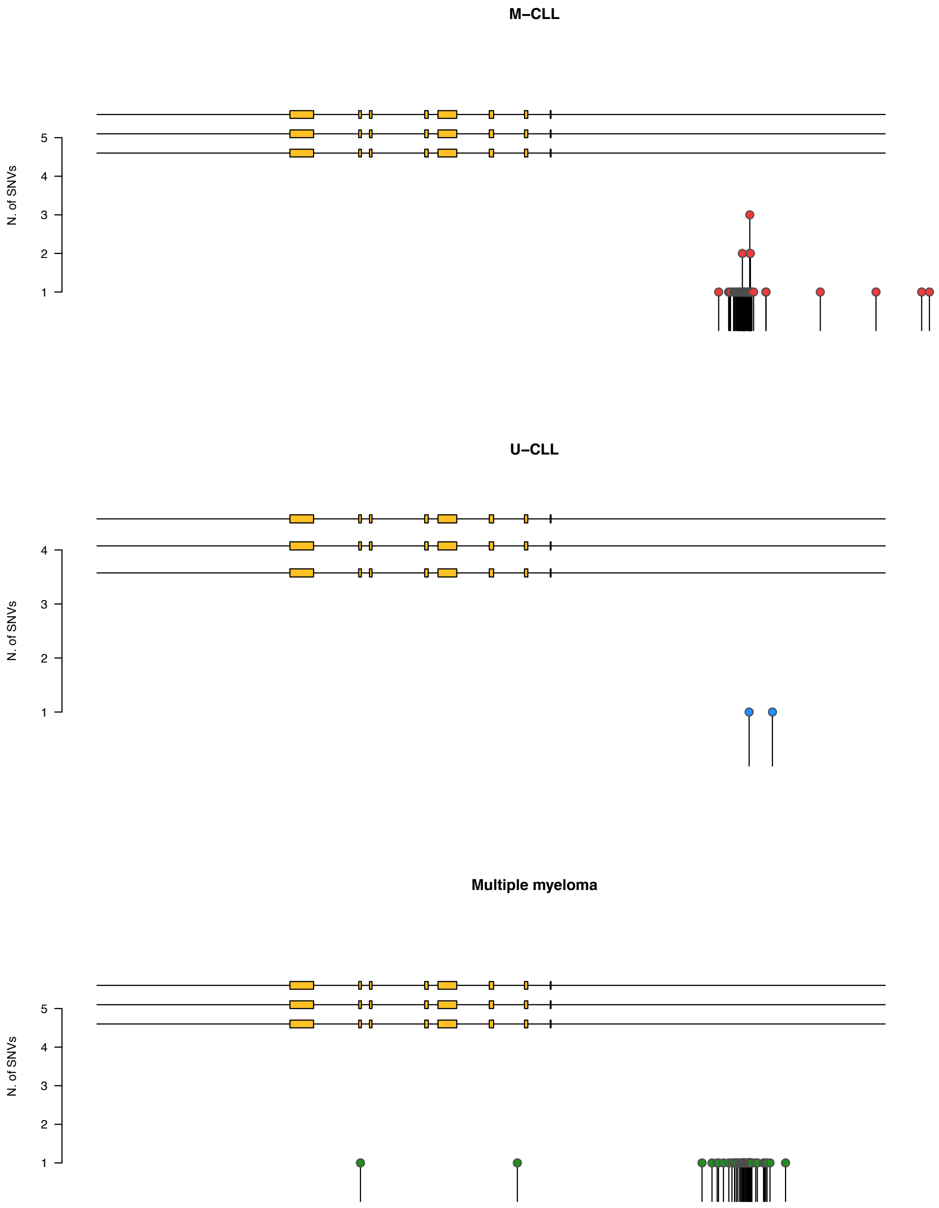

Supplementary Figure 11. Relative contribution of mutational signatures in CLL. Hierarchical clustering based on the relative contribution of each extracted mutational signature among 146 CLLs. Two main group are created, one with high and one with low signature 9 contribution. The first is mostly composed by M-CLL conversely the second grouped the great majority of U-CLL.

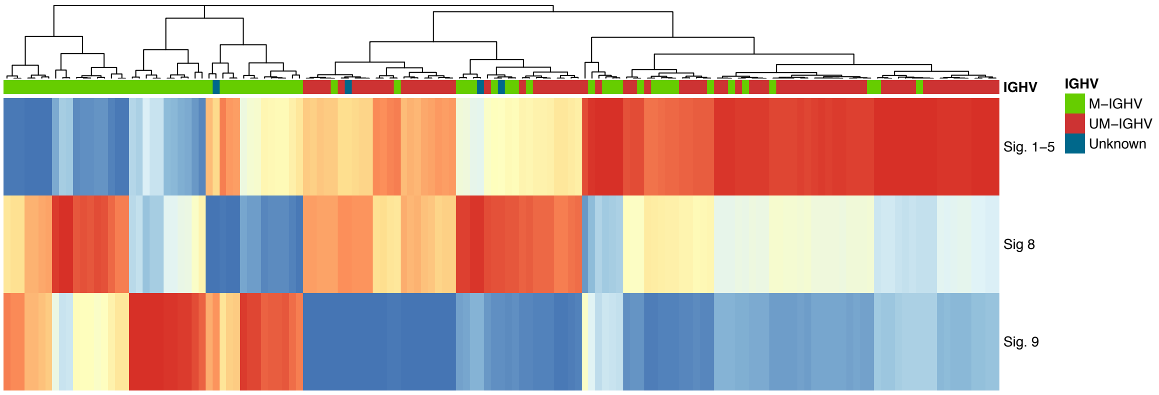

Supplementary Figure 12. The concordance rate between the published list of CLL variants on the IGH/IGK/IGL loci extracted by Sidron and the one extracted by 3 additional SNV callers (mutect2, caveman and muse).

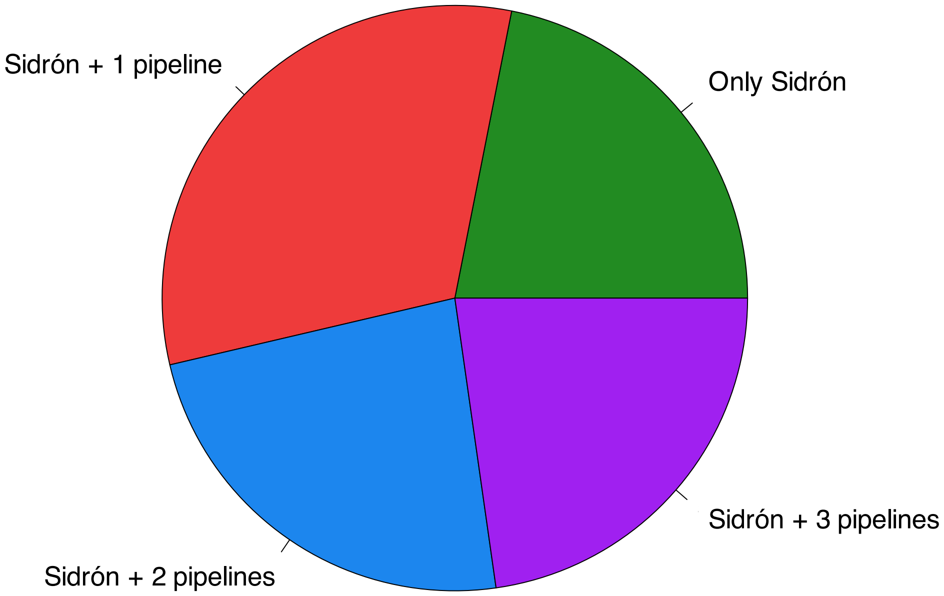

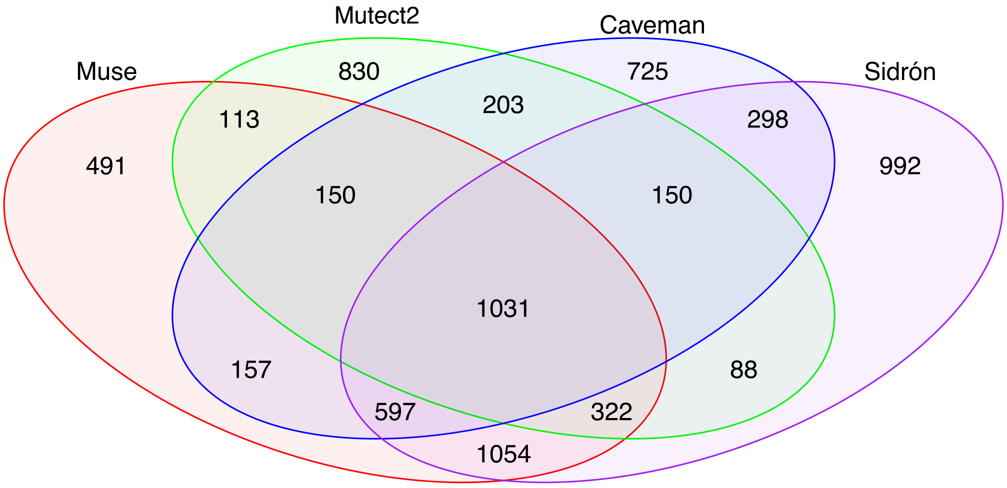

Supplementary References

1. Alexandrov, L., *et al.* The Repertoire of Mutational Signatures in Human Cancer. Preprint at https://www.biorxiv.org/content/10.1101/322859v1 2018)

2. Bolli, N., *et al.* Genomic patterns of progression in smoldering multiple myeloma. *Nat Commun* **9**, 3363 (2018).

3. Kasar, S., *et al.* Whole-genome sequencing reveals activation-induced cytidine deaminase signatures during indolent chronic lymphocytic leukaemia evolution. *Nat Commun* **6**, 8866 (2015).
