# Supplementary material for: A practical guide for mutational signature analysis in hematological malignancies
Source: Nat Commun. 2019 Jul 5;10:2969. doi: 10.1038/s41467-019-11037-8 (PMC6611883; doi:10.1038/s41467-019-11037-8)
Supplement: Supplementary file 3 — Description of Additional Supplementary Files [file 41467_2019_11037_MOESM3_ESM.pdf]

Description of Additional Supplementary Files

File Name: Supplementary Data 1
Description: Assignment of signatures. Each of the signatures extracted with either mutationalPatterns or the method from Alexandrov et al. were assigned to one or a combination of two COSMIC signatures. To do so, cosine similarities between the extracted signatures and each COSMIC signature, or a linear combination of two COSMIC signatures (using non-negative least squares R package NNLS), were computed.

File Name: Supplementary Data 2
Description: Summary of clinical and biological features of 143 chronic lymphocytic leukemia myeloma samples.

File Name: Supplementary Software 1
Description: R used to generate signature data for multiple myeloma genomes using mutationalPatterns in the paper

File Name: Supplementary Software 2
Description: R used to generate signature data for chronic lymphocytic leukemia genomes using mutationalPatterns in the paper

File Name: Supplementary Software 3
Description: R used to generate signature data for acute myeloid leukemia genomes using mutationalPatterns in the paper
