# Supplementary material for: A practical guide for mutational signature analysis in hematological malignancies
Source: Nat Commun. 2019 Jul 5;10:2969. doi: 10.1038/s41467-019-11037-8 (PMC6611883; doi:10.1038/s41467-019-11037-8)
Supplement: Supplementary file 6 — Supplementary Software 1 [file 41467_2019_11037_MOESM6_ESM.zip › 186038_2_data_set_3827833_pryddt (1).pdf]

Mutational Pattern Signature Analysis on Multiple Myeloma

Created by Francesco Maura (mauraf@mskcc.org)

Built with R version:
3.4.2

Libraries

Load necessary libraries

```
ref_genome <- "BSgenome.Hsapiens.UCSC.hg19"
# source("https://bioconductor.org/biocLite.R")
library("gridExtra")
library("MutationalPatterns")
```

```
## Loading required package: GenomicRanges
```

```
## Loading required package: stats4
```

```
## Loading required package: BiocGenerics
```

```
## Loading required package: parallel
```

```
##
## Attaching package: 'BiocGenerics'
```

```
## The following objects are masked from 'package:parallel':
##
##     clusterApply, clusterApplyLB, clusterCall, clusterEvalQ,
##     clusterExport, clusterMap, parApply, parCapply, parLapply,
##     parLapplyLB, parRapply, parSapply, parSapplyLB
```

```
## The following object is masked from 'package:gridExtra':
##
##     combine
```

```
## The following objects are masked from 'package:stats':
##
##     IQR, mad, sd, var, xtabs
```

```
## The following objects are masked from 'package:base':
##
##     anyDuplicated, append, as.data.frame, cbind, colMeans,
##     colnames, colSums, do.call, duplicated, eval, evalq, Filter,
##     Find, get, grep, grepl, intersect, is.unsorted, lapply,
##     lengths, Map, mapply, match, mget, order, paste, pmax,
##     pmax.int, pmin, pmin.int, Position, rank, rbind, Reduce,
##     rowMeans, rownames, rowSums, sapply, setdiff, sort, table,
##     tapply, union, unique, unsplit, which, which.max, which.min
```

```
## Loading required package: S4Vectors
```

```
##
## Attaching package: 'S4Vectors'
```

```
## The following object is masked from 'package:base':
##
##     expand.grid
```

```
## Loading required package: IRanges
```

```
## Loading required package: GenomeInfoDb
```

```
## Loading required package: NMF
```

```
## Loading required package: pkgmaker
```

```
## Loading required package: registry
```

```
##
## Attaching package: 'pkgmaker'
```

```
## The following object is masked from 'package:S4Vectors':
##
##     new2
```

```
## Loading required package: rngtools
```

```
## Loading required package: cluster
```

```
## NMF - BioConductor layer [OK] | Shared memory capabilities [NO: bigmemory] | Cores
3/4
```

```
##   To enable shared memory capabilities, try: install.extras('
## NMF
## ')
```

```
##
## Attaching package: 'NMF'
```

```
## The following object is masked from 'package:S4Vectors':
##
##     nrun
```

```
library(BSgenome.Hsapiens.UCSC.hg19)
```

```
## Loading required package: BSgenome
```

```
## Loading required package: Biostrings
```

```
## Loading required package: XVector
```

```
##
## Attaching package: 'Biostrings'
```

```
## The following object is masked from 'package:base':
##
##     strsplit
```

```
## Loading required package: rtracklayer
```

```
library(RColorBrewer)
library(GenomicRanges)
library(GenomicFeatures)
```

```
## Loading required package: AnnotationDbi
```

```
library(BSgenome)
library(ref_genome, character.only = TRUE)
library(BSgenome.Celegans.UCSC.ce2)
library("NMF")
```

```
cave_filter<- read.delim("first_MM_cases_30.txt", sep="\t", header=TRUE, stringsAsFac
tors = F)
cave_filter$chr<- paste("chr",cave_filter$chrom,sep="")

alfa = with(cave_filter, GRanges(chr, IRanges(start=pos, end=pos)))
# annotate meta cols
values(alfa) <- cave_filter[,c("sample","ref", "alt")]

types = mut_type(alfa)
context = mut_context(alfa, ref_genome)
type_context = type_context(alfa, ref_genome)

#### for each sample create GRange file in a list

g<- list()
list<- unique(cave_filter$sample)
for(i in (1:length(list)))
{
  cave_filter_single<- cave_filter[cave_filter$sample== list[i],]
  alfa_single<- with(cave_filter_single, GRanges(chr, IRanges(start=cave_filter_singl
e$pos, end=cave_filter_single$pos), REF= ref, ALT=alt))
  #write.table(cave_filter_single, sprintf("%s_cave.txt",list[i]), sep="\t", row.name
s = F, col.names = F, quote=F)
  names(alfa_single)<- cave_filter_single$name
  genome(alfa_single) <- "hg19"
  g[[i]]<- (alfa_single)
}


names(g) <- list

type_occurrences <- mut_type_occurrences(g, ref_genome) ##### plot 6 classes and CpG
 prevalence
p1 = plot_spectrum(type_occurrences)
p2 = plot_spectrum(type_occurrences, CT = TRUE)
p3 = plot_spectrum(type_occurrences, CT = TRUE, legend = FALSE)


grid.arrange(p1, p2, p3, ncol=3, widths=c(3,3,1.75))
```

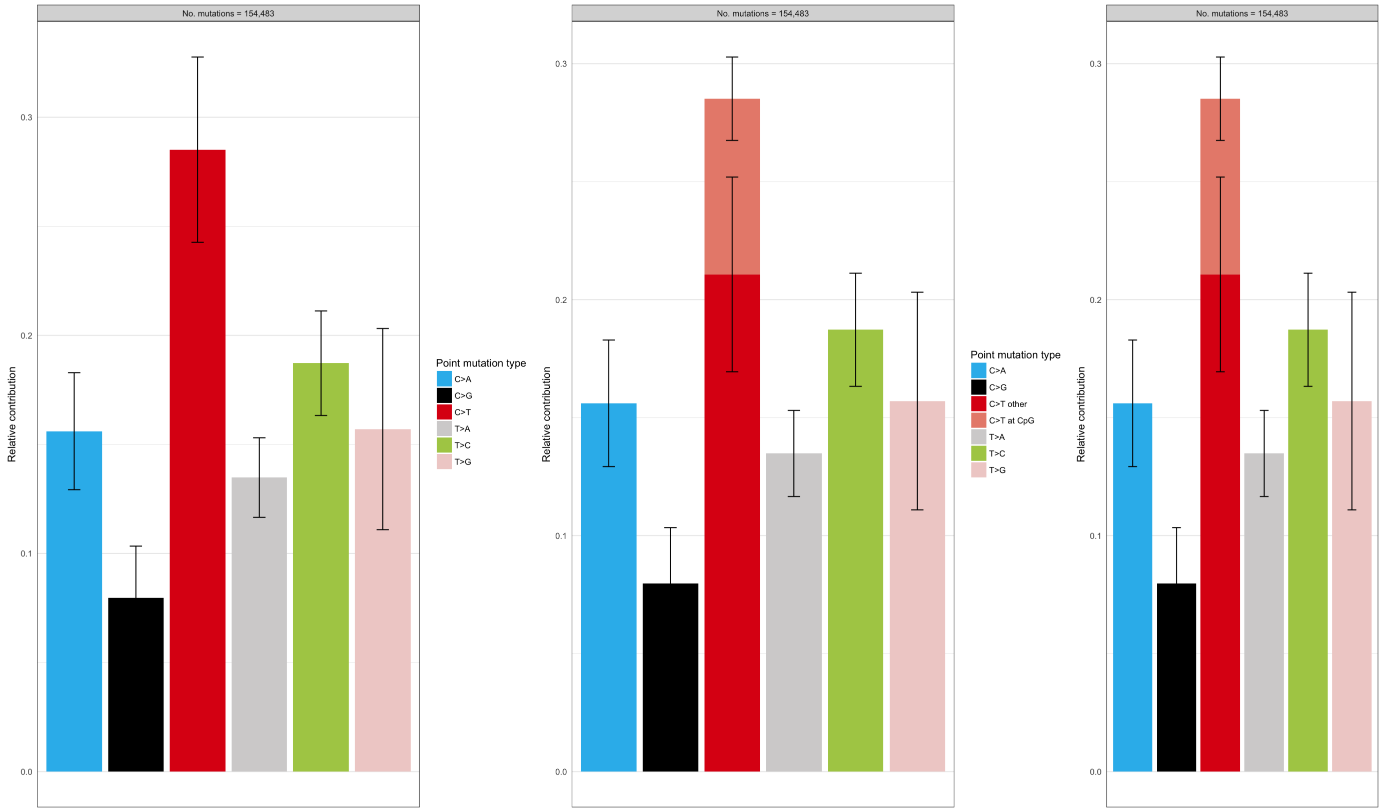


```
mut_mat <- mut_matrix(vcf_list = g, ref_genome = ref_genome)
plot_96_profile(mut_mat[,c(1,2)], ymax = 0.05) ############## plot 96 classes of firs
t 2 samples
```

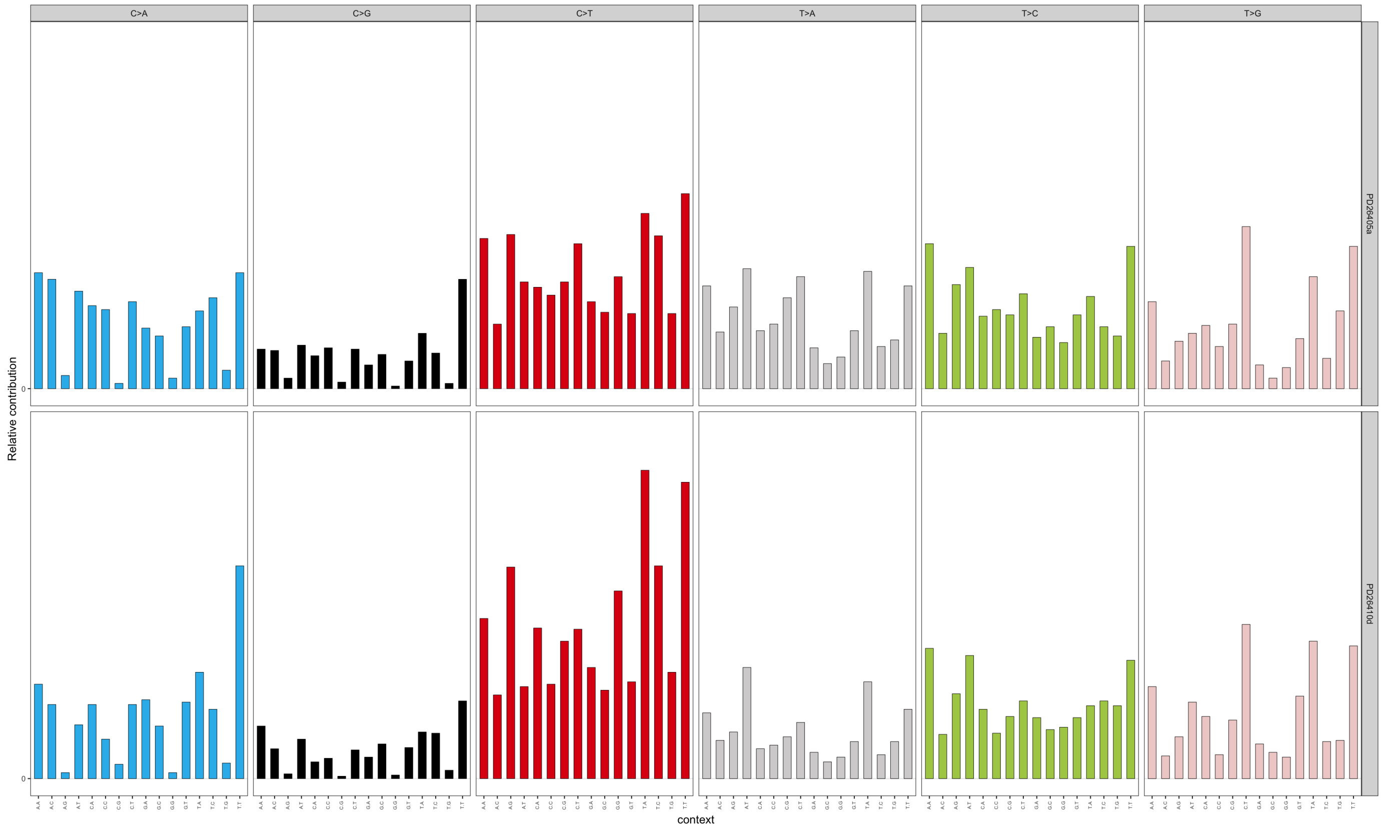


```
head(mut_mat)
```

```
##          PD26405a PD26410d PD26415c PD26426e PD26428a PD26401a PD26432c
## A[C>A]A        88       79       25      177       24      109       75
## A[C>A]C        83       62       20      139       17       70       62
## A[C>A]G        10        5        5       17        2        7        7
## A[C>A]T        74       45       23      129       28       65       43
## C[C>A]A        63       62       32      211       27       63       62
## C[C>A]C        60       33       12      131       13       49       44
##          PD26402a PD26411c PD26412a PD26414a PD26424a PD26425e PD26400a
## A[C>A]A        90       90      135       21       94      102      105
## A[C>A]C        61       60       99       18       71       67       83
## A[C>A]G        14       10       12        6       14       12       11
## A[C>A]T        85       66      102       17       79       50      100
## C[C>A]A        81      120      167       21       97       63       81
## C[C>A]C        60       39       92       11       66       49       73
##          PD26403a PD26427a PD26406a PD26408a PD26409a PD26422e PD26423e
## A[C>A]A        86       73       65       78       52       66      109
## A[C>A]C        76       59       49       63       53       62       84
## A[C>A]G         9       13       10       13        3        4       14
## A[C>A]T        67       56       45       63       46       50       56
## C[C>A]A        85       58       54       78       49       75      130
## C[C>A]C        56       49       31       55       55       33       42
##          PD26429a PD26434c PD26404a PD26419a PD26420a PD26416d PD26435c
## A[C>A]A        52       19       55       94       56       82      118
## A[C>A]C        51       12       37       76       50       56      104
## A[C>A]G         7        1       10       15        9       15       17
## A[C>A]T        31        8       38       87       50       40       92
## C[C>A]A        47       13       53       96       46       70       99
## C[C>A]C        29        4       45       72       58       40      100
##          PD26407a PD26418a
## A[C>A]A        32      111
## A[C>A]C        21       89
## A[C>A]G         5       13
## A[C>A]T        15      101
## C[C>A]A        36      110
## C[C>A]C        20       92
```

Run NNMF for signatures extraction

```
mut_mat = mut_mat + 0.0001
estimate = nmf(mut_mat, rank=2:6, method="brunet", nrun=1000, seed=123456)
plot(estimate)
```

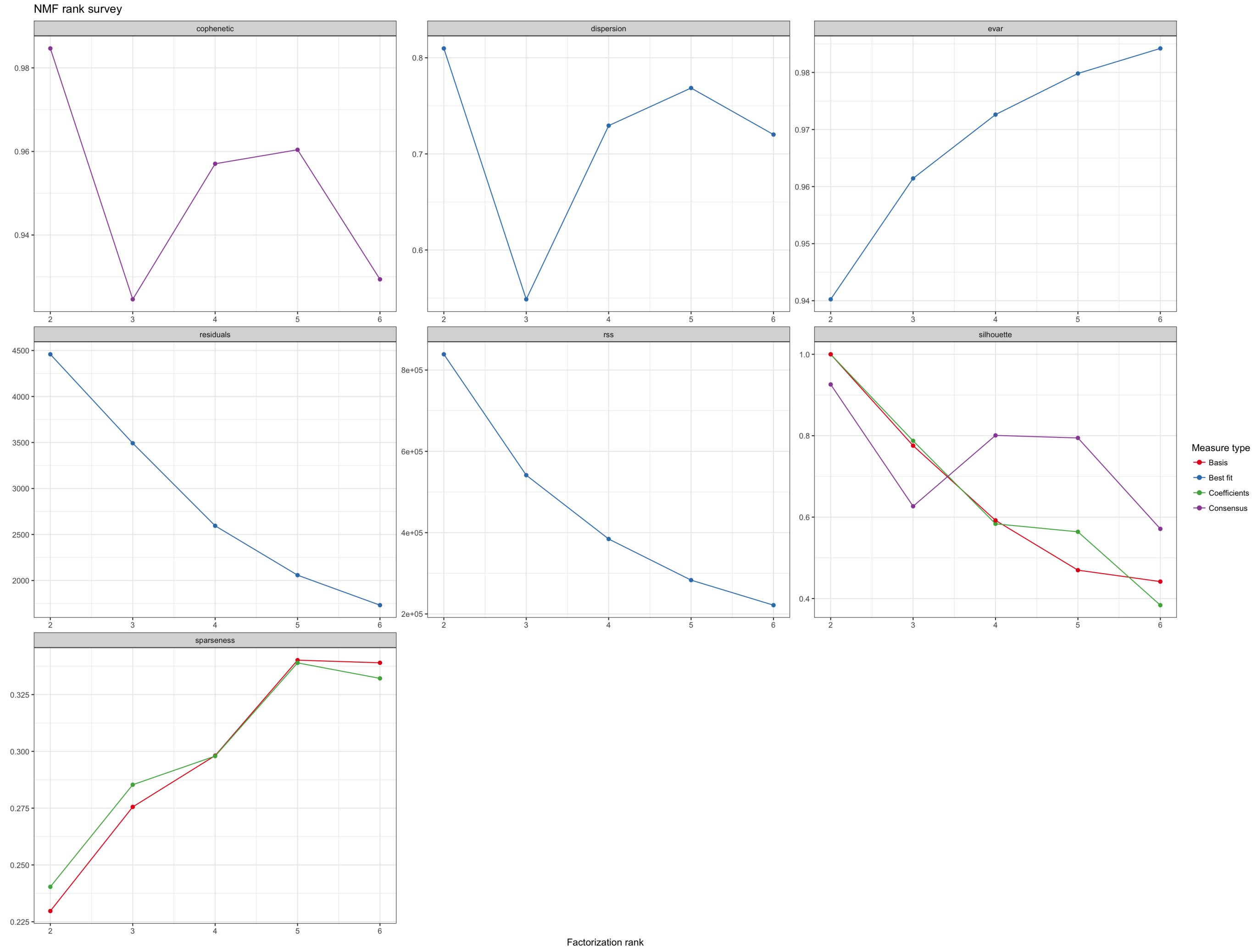


```
nmf_res <- extract_signatures(mut_mat, rank = 5) #### select best solution
colnames(nmf_res$signatures) <- c("Signature A", "Signature B",  "Signature C", "Sign
ature D", "Signature E")
head(nmf_res$signatures)
```

```
##          Signature A Signature B Signature C Signature D Signature E
## A[C>A]A   155.89442   396.79065   371.75545    376.9963   689.88302
## A[C>A]C   104.90040   299.21732   305.70059    284.7581   568.71936
## A[C>A]G    17.55335    34.50444    73.68538     39.7574    84.98353
## A[C>A]T   161.29963   111.69724   381.89240    199.8428   632.35744
## C[C>A]A    91.48014   344.61511   292.77798    561.3654   605.11443
## C[C>A]C    81.93030   151.85141   282.70817    129.2408   620.03740
```

```
plot_96_profile(nmf_res$signatures, ymax = 0.05)
```

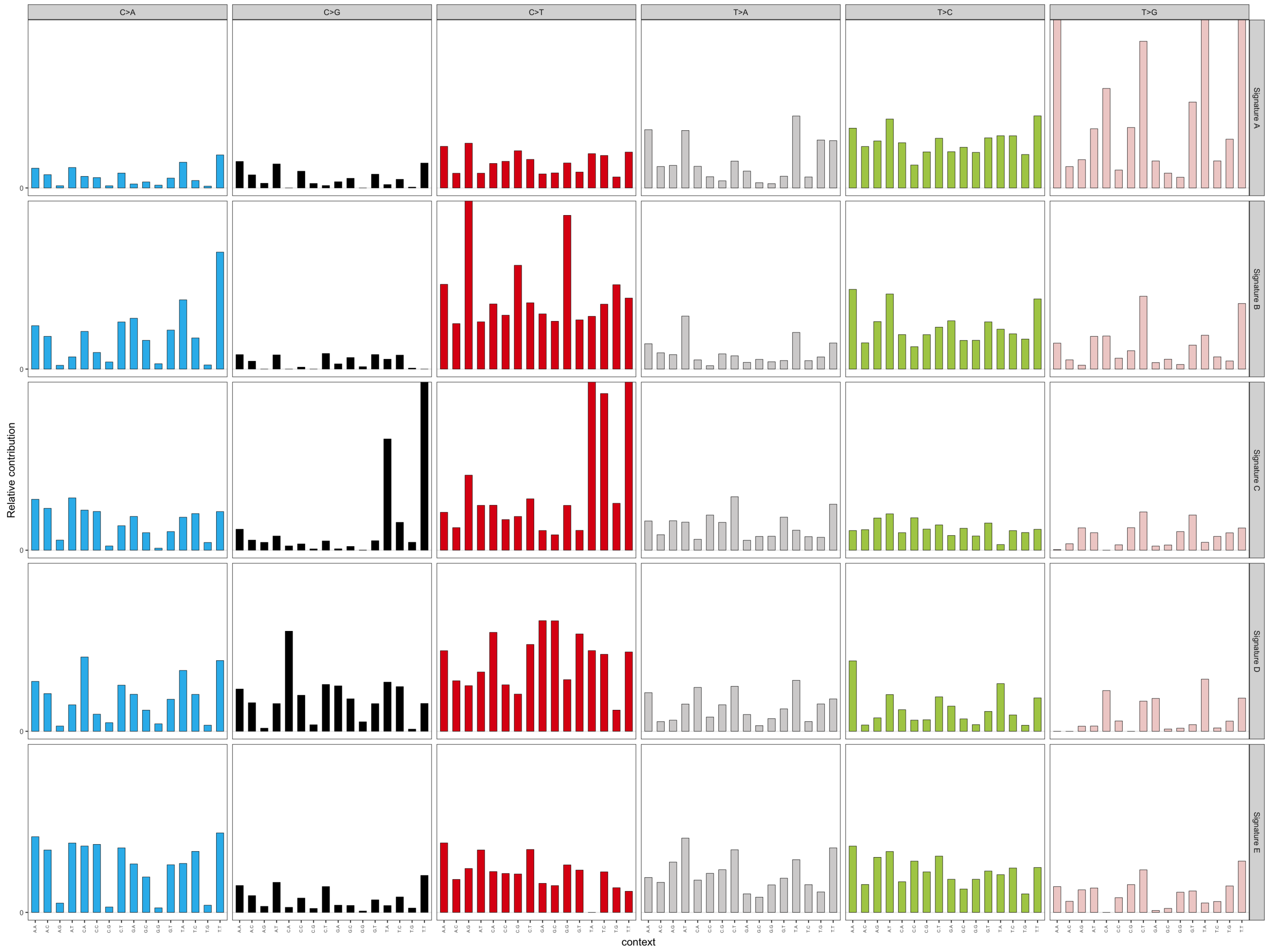


```
rownames(nmf_res$contribution) <- c("Signature A", "Signature B",  "Signature C", "Si
gnature D", "Signature E")

pc1 <- plot_contribution(nmf_res$contribution, nmf_res$signature, mode = "relative",
                         palette =c(brewer.pal(8,"Dark2")), coord_flip = TRUE)
pc2 <- plot_contribution(nmf_res$contribution, nmf_res$signature, mode = "absolute",
                         palette = c(brewer.pal(8,"Dark2")), coord_flip = TRUE)
grid.arrange(pc1, pc2)
```

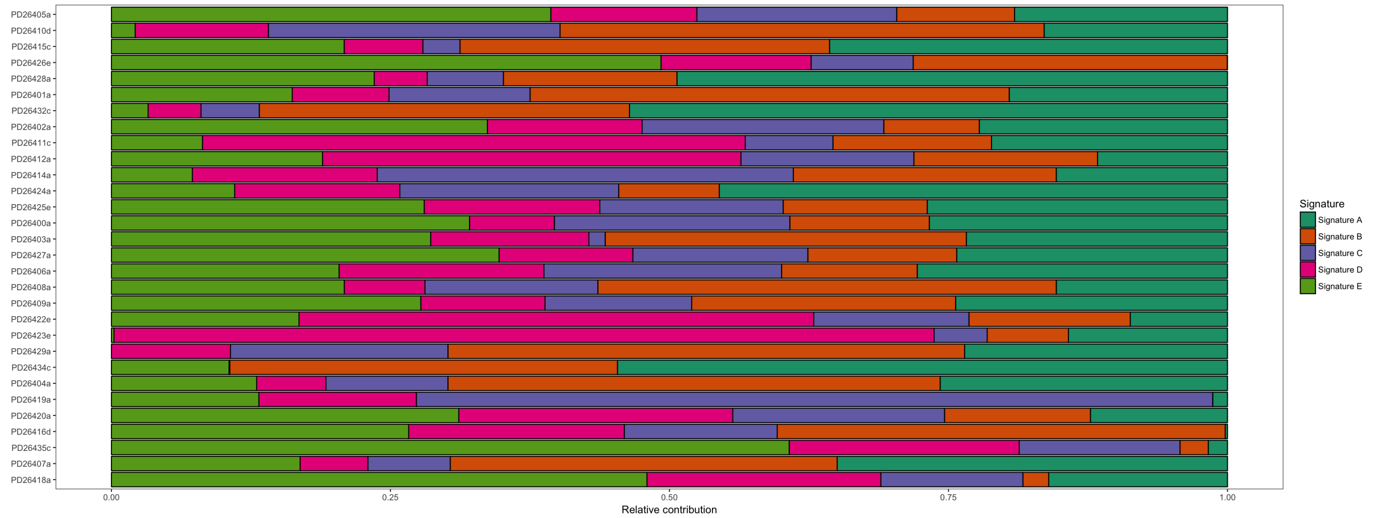

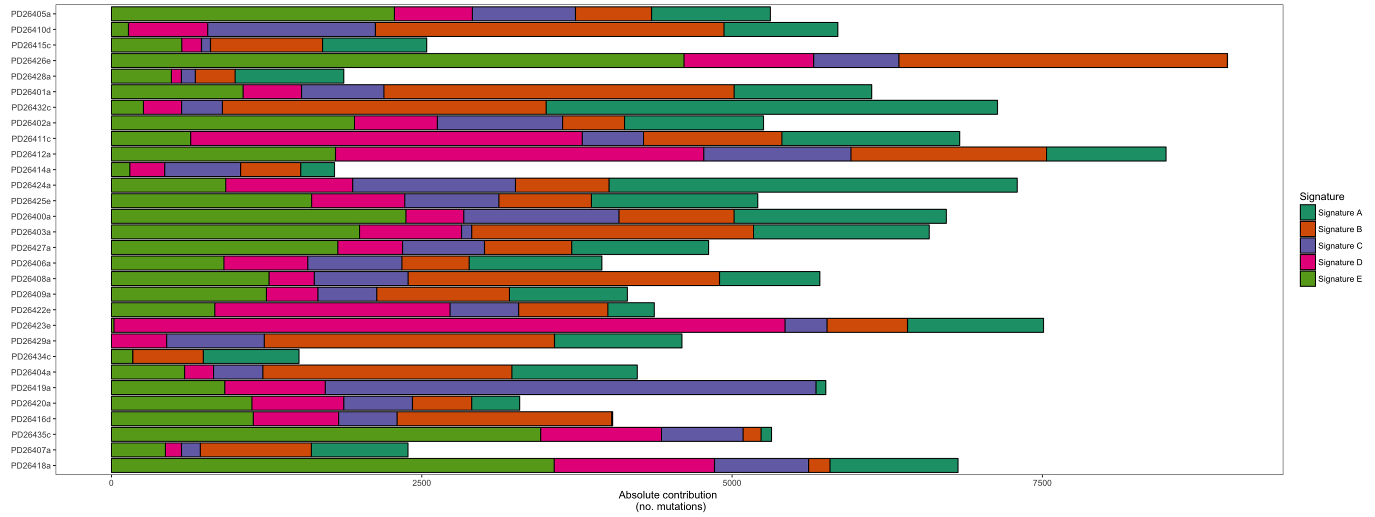


```
#### generate heatmap for patients clsutering

pch1 <- plot_contribution_heatmap(nmf_res$contribution)
pch2 <- plot_contribution_heatmap(nmf_res$contribution, cluster_samples=FALSE )
grid.arrange(pch1, pch2, ncol = 2, widths = c(2,1.6))
```

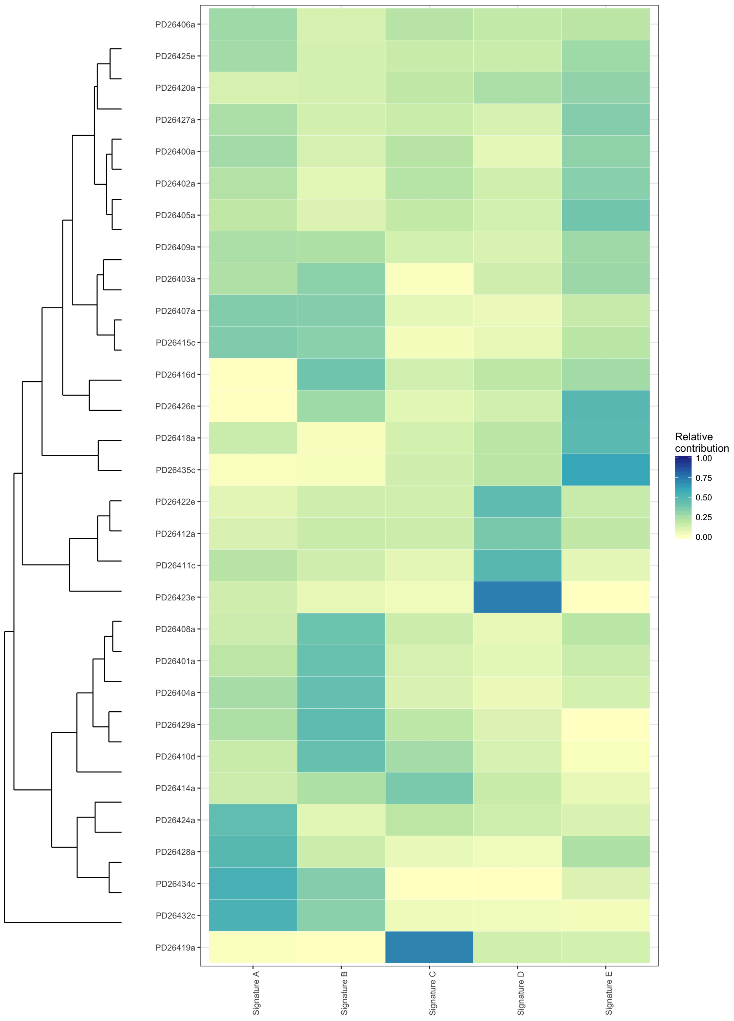

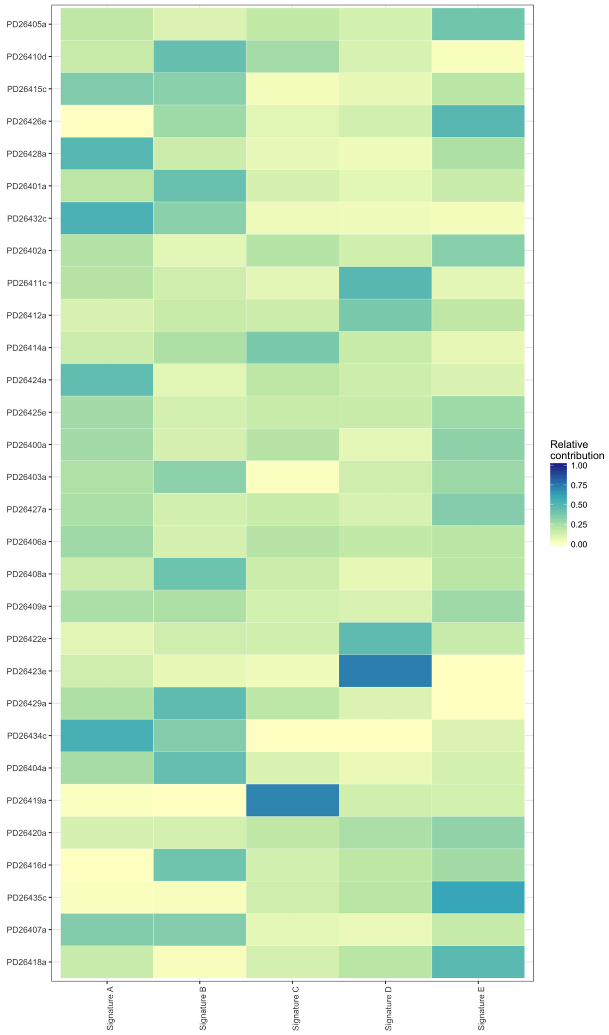


```
#### Compare the reconstructed mutational profile with the original mutational profil
e:

plot_compare_profiles(mut_mat[,1],
                      nmf_res$reconstructed[,1],
                      profile_names = c("Original", "Reconstructed"))
```

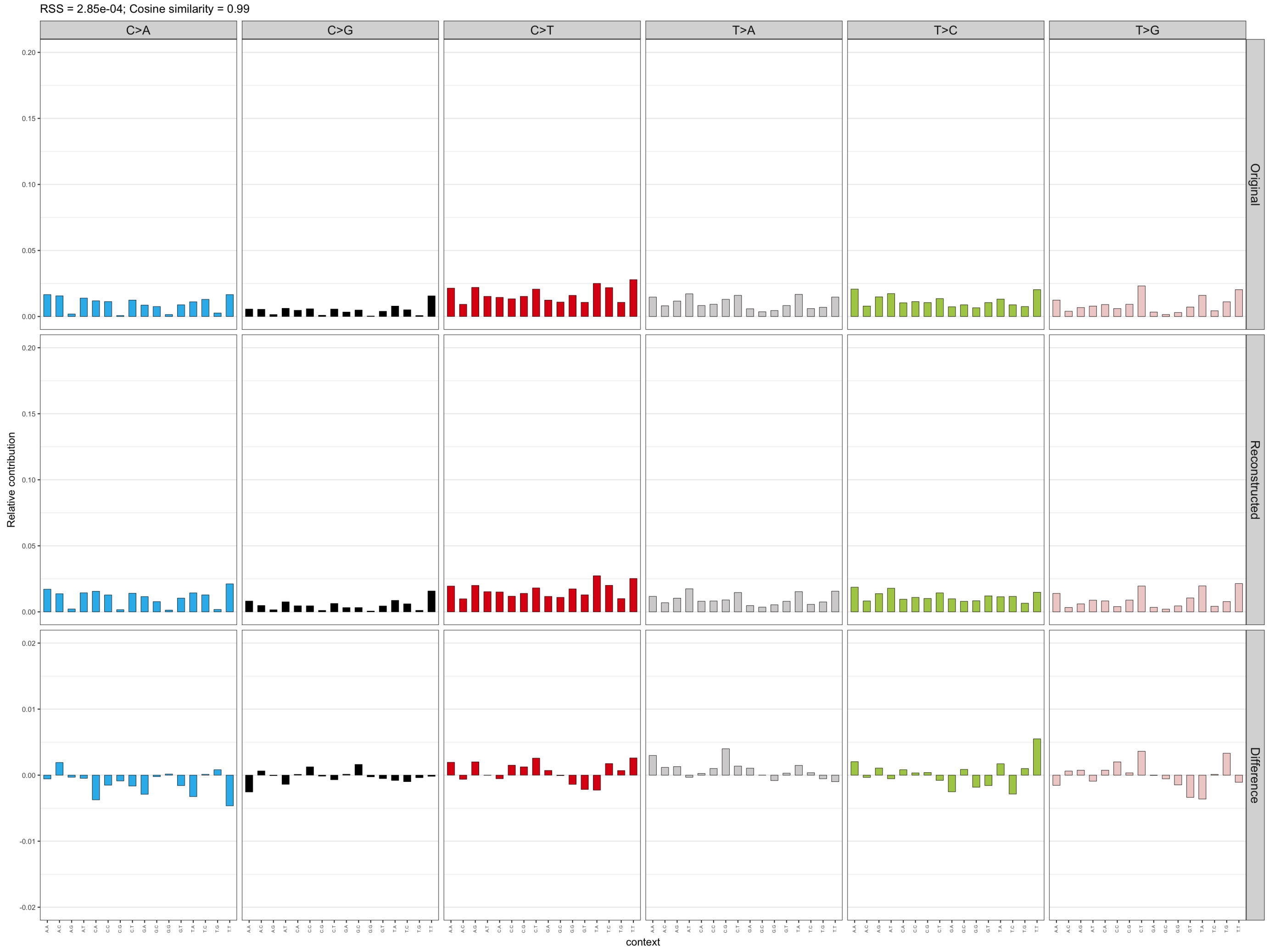

Upload 30 Signature COSMIC catalogue for fitting part

```
sp_url <- paste("http://cancer.sanger.ac.uk/cancergenome/assets/", "signatures_proba
bilities.txt", sep = "")
cancer_signatures = read.table(sp_url, sep = "\t", header = TRUE)

# Match the order of the mutation types to MutationalPatterns standard
new_order = match(row.names(mut_mat), cancer_signatures$Somatic.Mutation.Type)
# Reorder cancer signatures dataframe
cancer_signatures = cancer_signatures[as.vector(new_order),]
# Add trinucletiode changes names as row.names
row.names(cancer_signatures) = cancer_signatures$Somatic.Mutation.Type
# Keep only 96 contributions of the signatures in matrix
cancer_signatures = as.matrix(cancer_signatures[,4:33]) ###### all COSMIC Signatures
(columns signatures, rows 96 classes)
```

Fitting with all 30 COSMIC

```
mut_mat[,1:ncol(mut_mat)] = apply(mut_mat[,1:ncol(mut_mat)], 2, function(x) as.numeric(as.character(x)))
cancer_signatures[,1:ncol(cancer_signatures)] = apply(cancer_signatures[,1:ncol(cancer_signatures)], 2, function(x) as.numeric(as.character(x)))

fit_res <- fit_to_signatures(mut_mat, as.matrix(cancer_signatures))

plot_contribution(fit_res$contribution,
                  cancer_signatures[,1:ncol(cancer_signatures)],
                  coord_flip = TRUE,
                  mode = "absolute",
                  palette = c(brewer.pal(12,"Paired"), brewer.pal(8,"Dark2"),brewer.pal(11,"Spectral")))
```

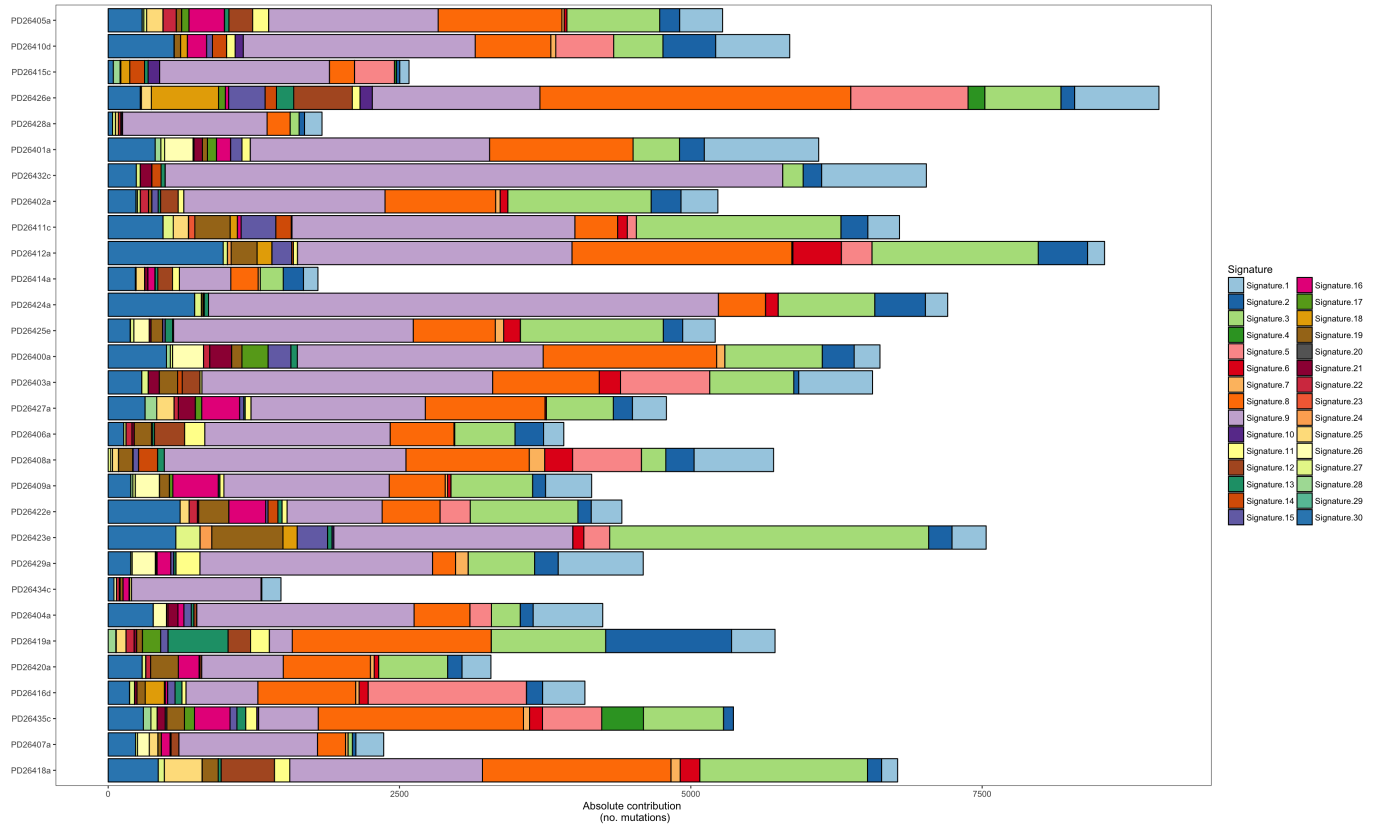

Fitting with COSMIC signatures extracted by NNMF

```
 cancer_signatures_selected <- as.data.frame.matrix(cancer_signatures[,c(1,2,5,8,9,13
)])

 ### include new signature in the fitting catalogue
 cancer_signatures_selected$MM1<- as.numeric(nmf_res$signatures[,4])/sum(nmf_res$sign
atures[,4])

 mut_mat[,1:ncol(mut_mat)] = apply(mut_mat[,1:ncol(mut_mat)], 2, function(x) as.numer
ic(as.character(x)))
 cancer_signatures_selected[,1:ncol(cancer_signatures_selected)] = apply(cancer_signa
tures_selected[,1:ncol(cancer_signatures_selected)], 2,
                                                                function(x) as.numeric(as.char
acter(x)))

 fit_res <- fit_to_signatures(mut_mat, as.matrix(cancer_signatures_selected))
 barplot((fit_res$contribution), las=2, col=c(brewer.pal(8,"Dark2")))
```

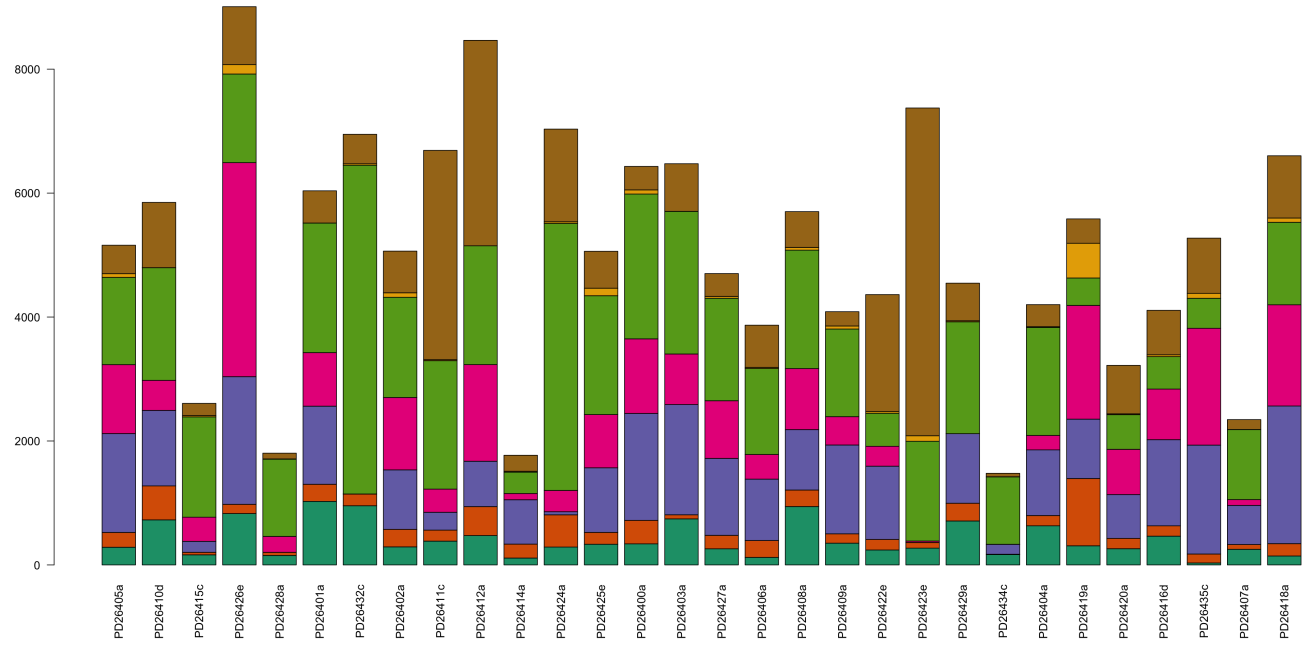


```
 plot_contribution(fit_res$contribution,
                   cancer_signatures[,1:ncol(cancer_signatures)],
                   coord_flip = T,
                   mode = "absolute", palette = c(brewer.pal(8,"Dark2")))
```

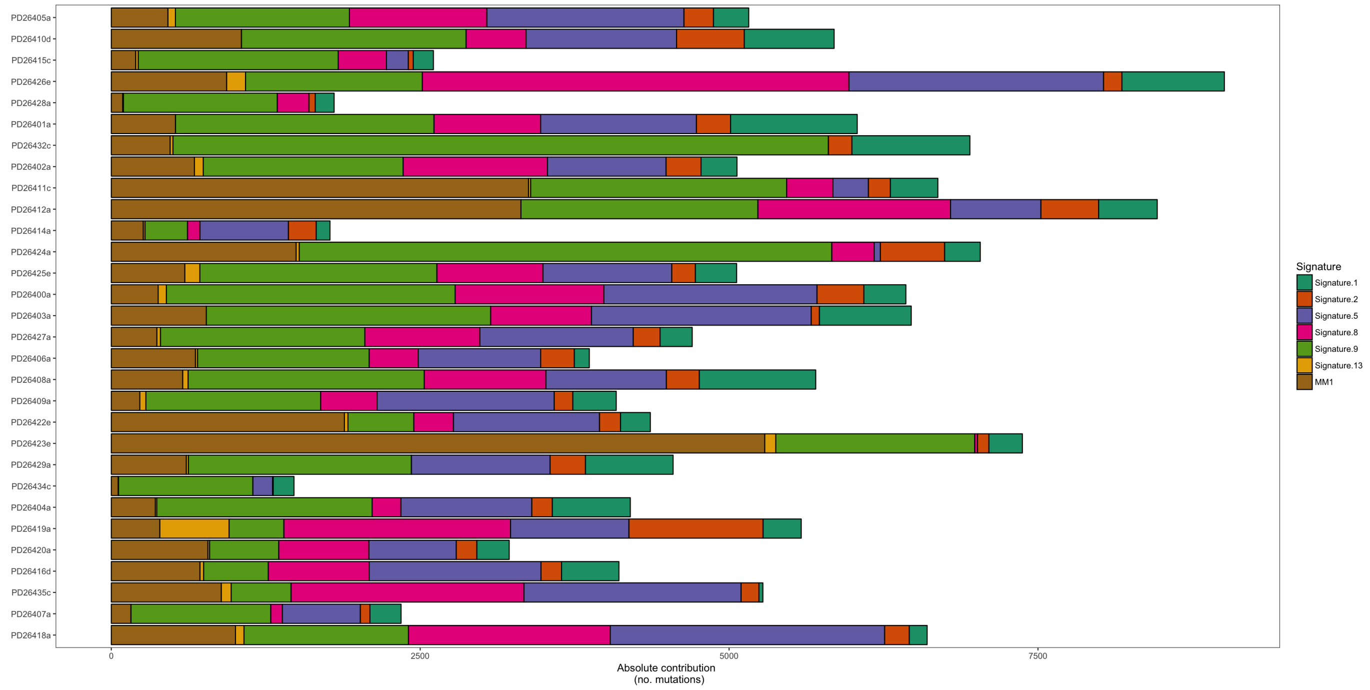

Note that the `echo = FALSE` parameter was added to the code chunk to prevent printing of the R code that generated the plot.
