# Supplementary material for: A practical guide for mutational signature analysis in hematological malignancies
Source: Nat Commun. 2019 Jul 5;10:2969. doi: 10.1038/s41467-019-11037-8 (PMC6611883; doi:10.1038/s41467-019-11037-8)
Supplement: Supplementary file 7 — Supplementary Software 2 [file 41467_2019_11037_MOESM7_ESM.zip › 186038_2_data_set_3827834_prydht.pdf]

Mutational Pattern Signature analysis on Chronic Lymphocytic Leukemia

Created by Francesco Maura (mauraf@mskcc.org)

Built with R version:
3.4.2

Libraries

Load necessary libraries

```
source("https://bioconductor.org/biocLite.R")
```

```
## Bioconductor version 3.6 (BiocInstaller 1.28.0), ?biocLite for help
```

```
## A new version of Bioconductor is available after installing the most
##   recent version of R; see http://bioconductor.org/install
```

```
library("gridExtra")
library("MutationalPatterns")
```

```
## Loading required package: GenomicRanges
```

```
## Loading required package: stats4
```

```
## Loading required package: BiocGenerics
```

```
## Loading required package: parallel
```

```
##
## Attaching package: 'BiocGenerics'
```

```
## The following objects are masked from 'package:parallel':
##
##     clusterApply, clusterApplyLB, clusterCall, clusterEvalQ,
##     clusterExport, clusterMap, parApply, parCapply, parLapply,
##     parLapplyLB, parRapply, parSapply, parSapplyLB
```

```
## The following object is masked from 'package:gridExtra':
##
##     combine
```

```
## The following objects are masked from 'package:stats':
##
##     IQR, mad, sd, var, xtabs
```

```
## The following objects are masked from 'package:base':
##
##     anyDuplicated, append, as.data.frame, cbind, colMeans,
##     colnames, colSums, do.call, duplicated, eval, evalq, Filter,
##     Find, get, grep, grepl, intersect, is.unsorted, lapply,
##     lengths, Map, mapply, match, mget, order, paste, pmax,
##     pmax.int, pmin, pmin.int, Position, rank, rbind, Reduce,
##     rowMeans, rownames, rowSums, sapply, setdiff, sort, table,
##     tapply, union, unique, unsplit, which, which.max, which.min
```

```
## Loading required package: S4Vectors
```

```
##
## Attaching package: 'S4Vectors'
```

```
## The following object is masked from 'package:base':
##
##     expand.grid
```

```
## Loading required package: IRanges
```

```
## Loading required package: GenomeInfoDb
```

```
## Loading required package: NMF
```

```
## Loading required package: pkgmaker
```

```
## Loading required package: registry
```

```
##
## Attaching package: 'pkgmaker'
```

```
## The following object is masked from 'package:S4Vectors':
##
##     new2
```

```
## Loading required package: rngtools
```

```
## Loading required package: cluster
```

```
## NMF - BioConductor layer [OK] | Shared memory capabilities [NO: bigmemory] | Cores
3/4
```

```
##   To enable shared memory capabilities, try: install.extras('
## NMF
## ')
```

```
##
## Attaching package: 'NMF'
```

```
## The following object is masked from 'package:S4Vectors':
##
##     nrun
```

```
library("BSgenome.Hsapiens.UCSC.hg19")
```

```
## Loading required package: BSgenome
```

```
## Loading required package: Biostrings
```

```
## Loading required package: XVector
```

```
##
## Attaching package: 'Biostrings'
```

```
## The following object is masked from 'package:base':
##
##     strsplit
```

```
## Loading required package: rtracklayer
```

```
library(RColorBrewer)
library(GenomicRanges)
library(GenomicFeatures)
```

```
## Loading required package: AnnotationDbi
```

```
library(BSgenome)
# library(ref_genome, character.only = TRUE)
library(BSgenome.Celegans.UCSC.ce2)
library("NMF")
ref_genome <- "BSgenome.Hsapiens.UCSC.hg19"
```

```
setwd("/Volumes/fm6/brca_mm_signature/mutational_pattern_for_MM///")
cave_filter<- read.delim("CLL_mutations.txt", sep="\t", header=T, stringsAsFactors =
F)
colnames(cave_filter)<- c("sample","chrom","pos","ref","alt")
cave_filter$chr<- paste("chr",cave_filter$chrom,sep="")

alfa = with(cave_filter, GRanges(chr, IRanges(start=pos, end=pos)))
# annotate meta cols
values(alfa) <- cave_filter[,c("sample","ref", "alt")]

types = mut_type(alfa)
context = mut_context(alfa, ref_genome)
type_context = type_context(alfa, ref_genome)

#### for each sample create GRange file in a list

g<- list()
list<- unique(cave_filter$sample)
for(i in (1:length(list)))
{
  cave_filter_single<- cave_filter[cave_filter$sample== list[i],]
  alfa_single<- with(cave_filter_single, GRanges(chr, IRanges(start=cave_filter_singl
e$pos, end=cave_filter_single$pos), REF= ref, ALT=alt))
  #write.table(cave_filter_single, sprintf("%s_cave.txt",list[i]), sep="\t", row.name
s = F, col.names = F, quote=F)
  names(alfa_single)<- cave_filter_single$name
  g[[i]]<- (alfa_single)
}


names(g) <- list

type_occurrences <- mut_type_occurrences(g, ref_genome) ##### plot 6 classes and CpG
 prevalence
p1 = plot_spectrum(type_occurrences)
p2 = plot_spectrum(type_occurrences, CT = TRUE)
p3 = plot_spectrum(type_occurrences, CT = TRUE, legend = FALSE)


grid.arrange(p1, p2, p3, ncol=3, widths=c(3,3,1.75))
```

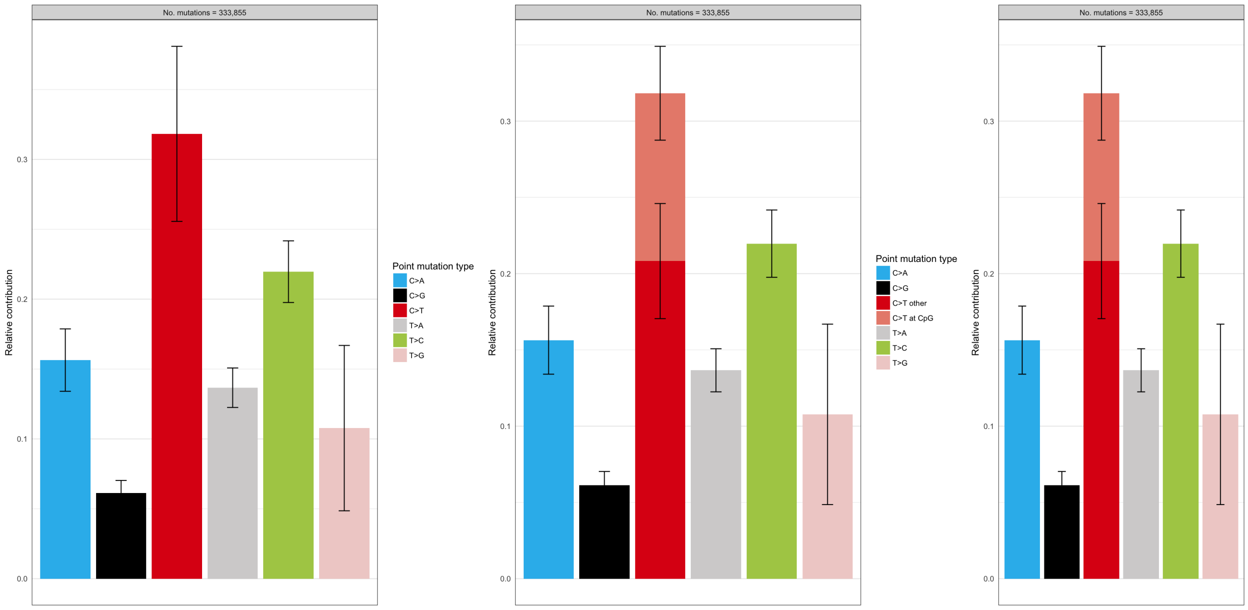


```
mut_mat <- mut_matrix(vcf_list = g, ref_genome = ref_genome)
plot_96_profile(mut_mat[,c(1,2)], ymax = 0.05) ############## plot 96 classes of firs
t 2 samples
```

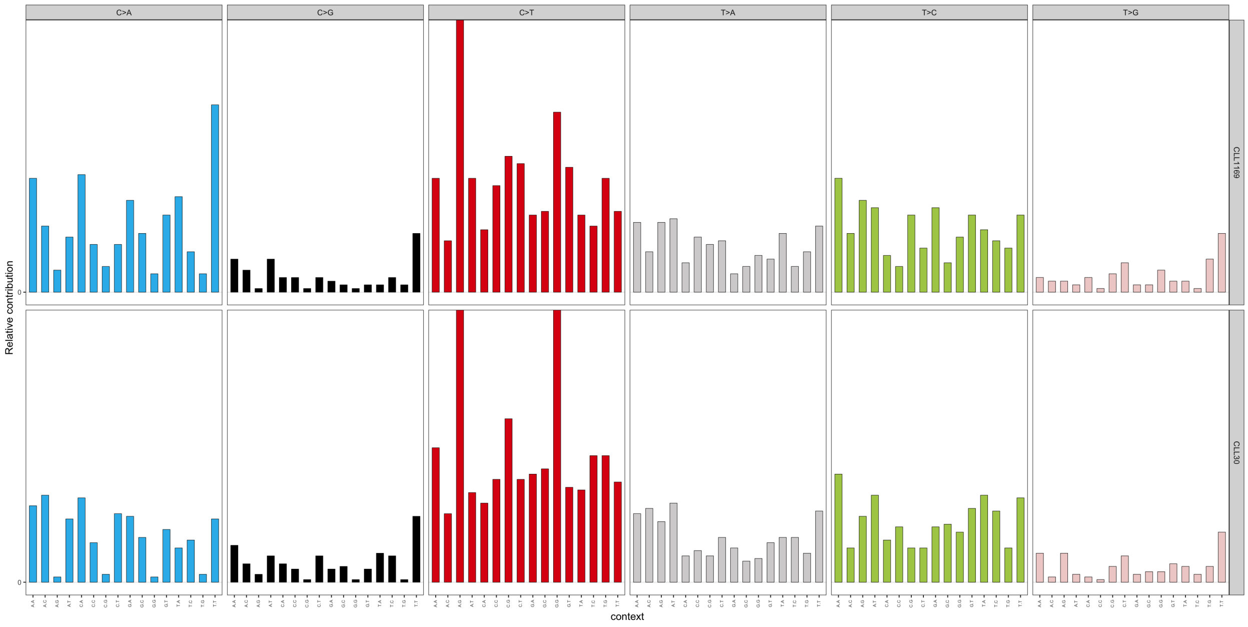

Run NNMF for signatures extraction

```
mut_mat = mut_mat + 0.0001
estimate = nmf(mut_mat, rank=2:6, method="brunet", nrun=10, seed=123456)
plot(estimate)
```

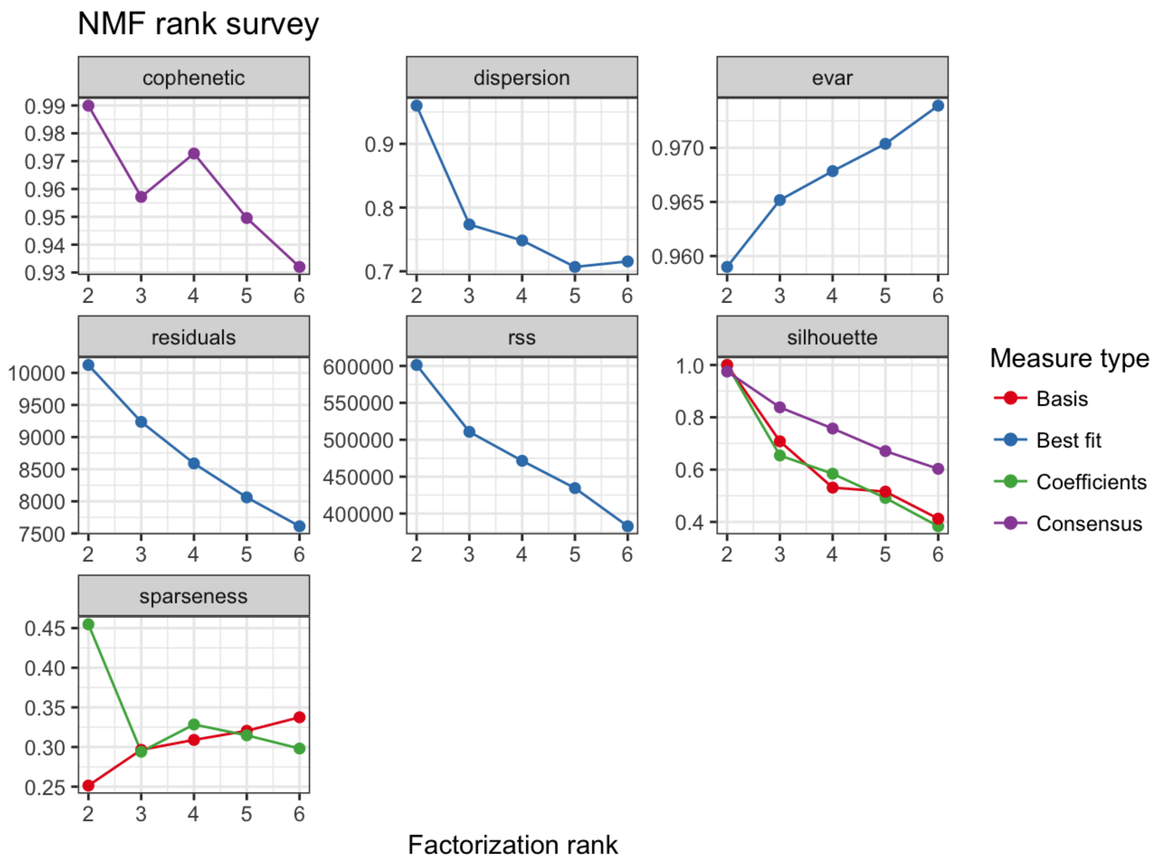


```
nmf_res <- extract_signatures(mut_mat, rank = 3) #### select best solution
colnames(nmf_res$signatures) <- c("Signature A", "Signature B",  "Signature C")

plot_96_profile(nmf_res$signatures, ymax = 0.05)
```

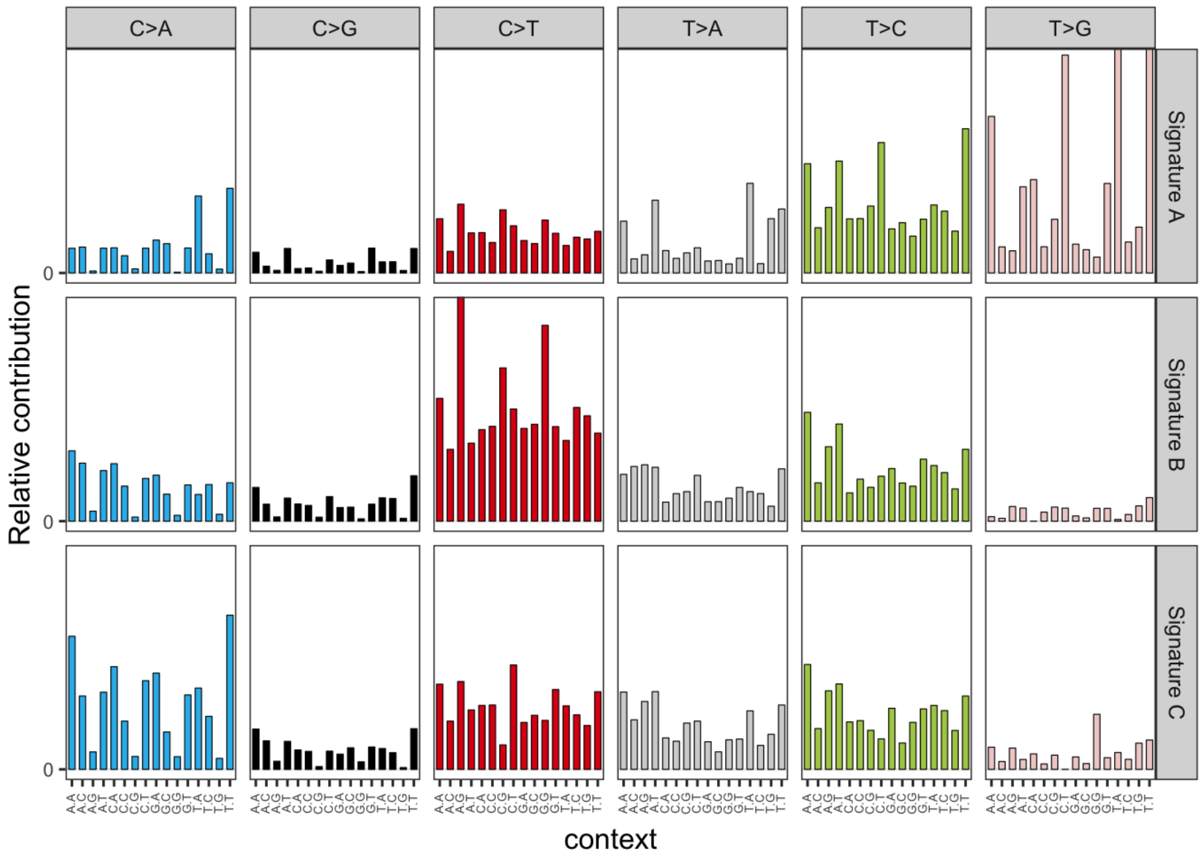


```
rownames(nmf_res$contribution) <- c("Signature A", "Signature B",  "Signature C")
pc1 <- plot_contribution(nmf_res$contribution, nmf_res$signature, mode = "relative")
pc2 <- plot_contribution(nmf_res$contribution, nmf_res$signature, mode = "absolute")
grid.arrange(pc1, pc2)
```

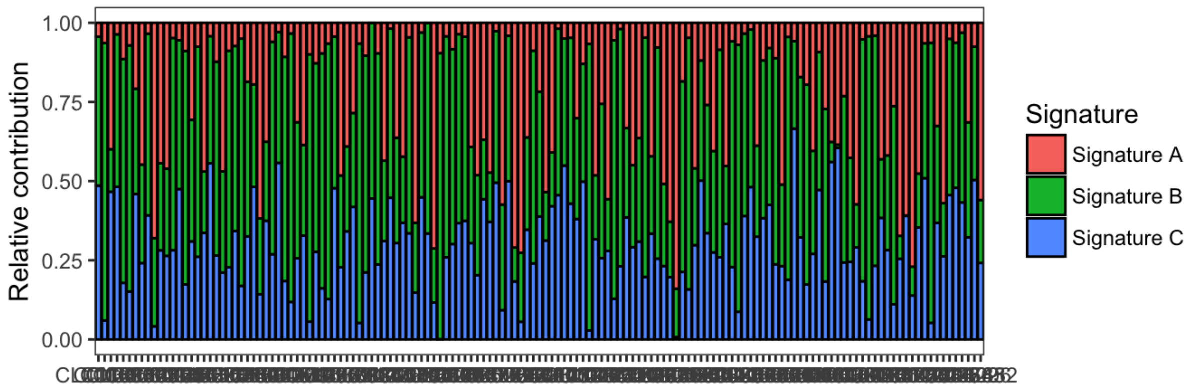

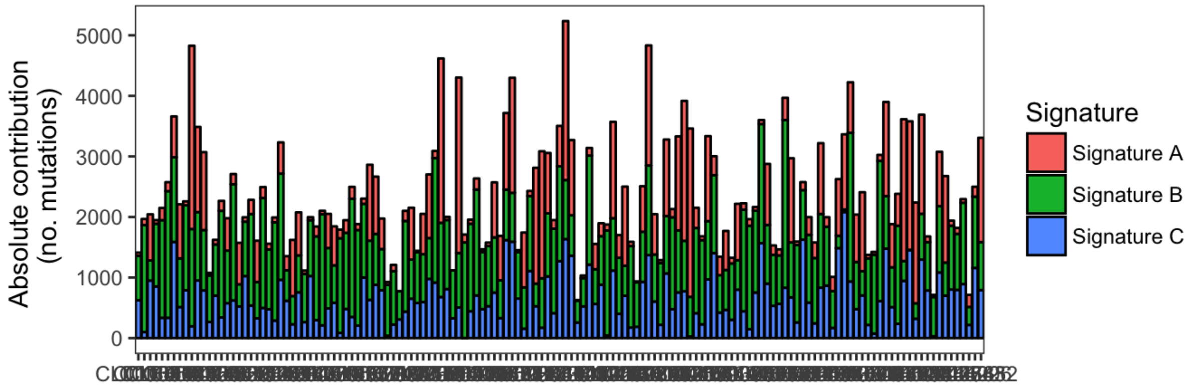


```
#### generate heatmap for patients clsutering

pch1 <- plot_contribution_heatmap(nmf_res$contribution)
pch2 <- plot_contribution_heatmap(nmf_res$contribution, cluster_samples=FALSE)
grid.arrange(pch1, pch2, ncol = 2, widths = c(2,1.6))
```

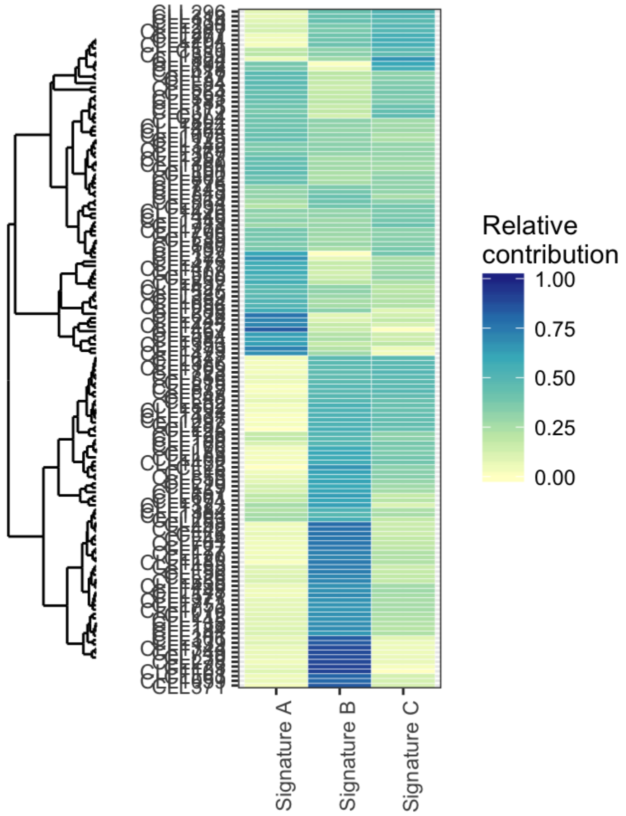

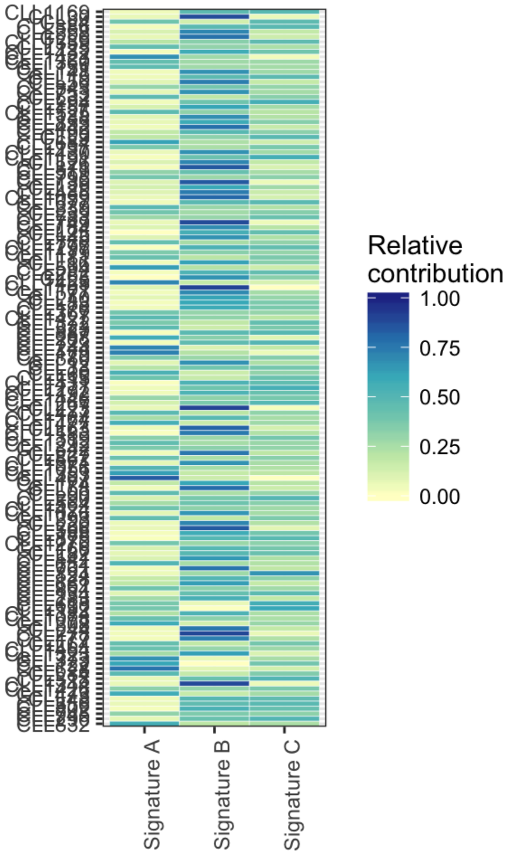


```
#### Compare the reconstructed mutational profile with the original mutational profil
e:

plot_compare_profiles(mut_mat[,1],
                      nmf_res$reconstructed[,1],
                      profile_names = c("Original", "Reconstructed"))
```

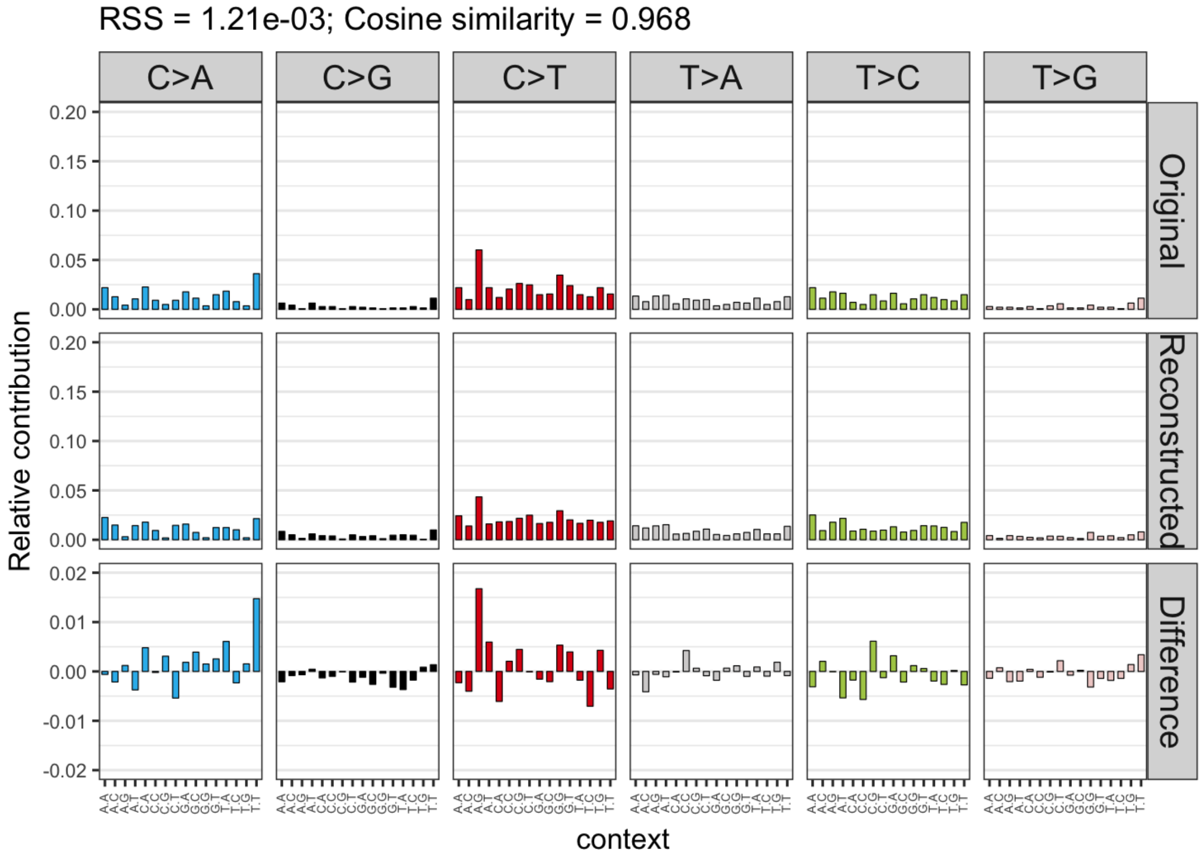

Upload 30 Signature COSMIC catalogue for fitting part

```
sp_url <- paste("http://cancer.sanger.ac.uk/cancergenome/assets/", "signatures_proba
bilities.txt", sep = "")
cancer_signatures = read.table(sp_url, sep = "\t", header = TRUE)
# Match the order of the mutation types to MutationalPatterns standard
new_order = match(row.names(mut_mat), cancer_signatures$Somatic.Mutation.Type)
# Reorder cancer signatures dataframe
cancer_signatures = cancer_signatures[as.vector(new_order),]
# Add trinucletiode changes names as row.names
row.names(cancer_signatures) = cancer_signatures$Somatic.Mutation.Type
# Keep only 96 contributions of the signatures in matrix
cancer_signatures = as.matrix(cancer_signatures[,4:33]) ###### all COSMIC Signatures
(columns signatures, rows 96 classes)
```

Fitting with all 30 COSMIC

```
 mut_mat[,1:ncol(mut_mat)] = apply(mut_mat[,1:ncol(mut_mat)], 2, function(x) as.numer
ic(as.character(x)))
 cancer_signatures[,1:ncol(cancer_signatures)] = apply(cancer_signatures[,1:ncol(canc
er_signatures)], 2, function(x) as.numeric(as.character(x)))

 fit_res <- fit_to_signatures(mut_mat, as.matrix(cancer_signatures))
 plot_contribution(fit_res$contribution,
                   cancer_signatures[,1:ncol(cancer_signatures)],
                   coord_flip = FALSE,
                   mode = "absolute")
```

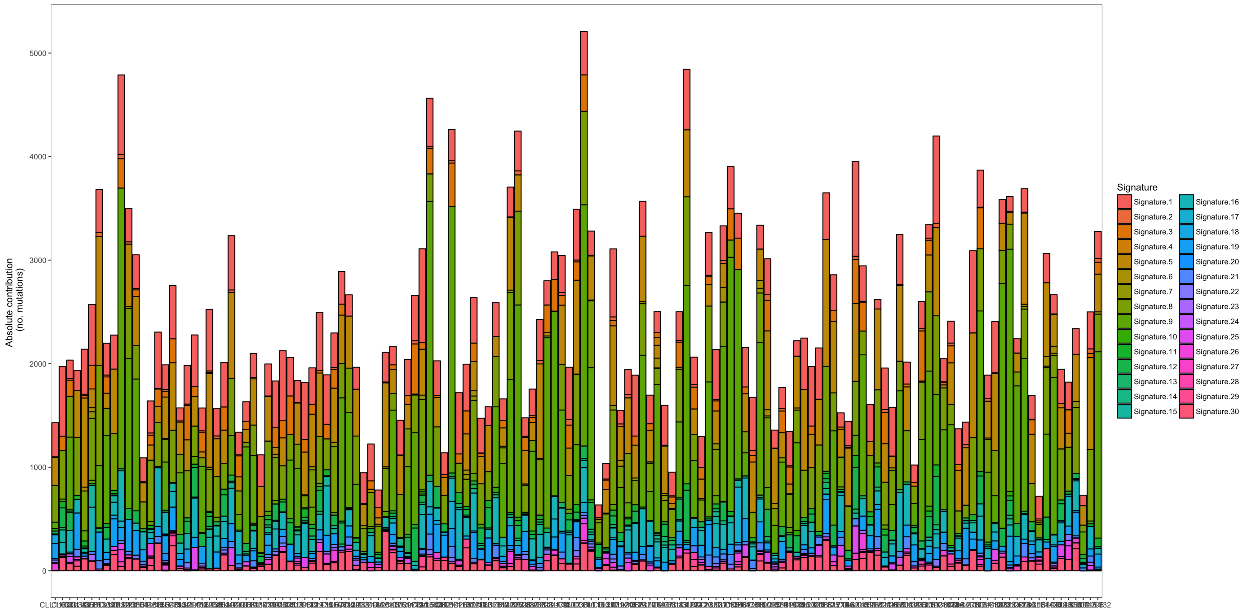

Fitting with COSMIC signatures extracted by NNMF

```
 cancer_signatures_selected <- as.data.frame.matrix(cancer_signatures[,c(1,5,8,9)])
 mut_mat[,1:ncol(mut_mat)] = apply(mut_mat[,1:ncol(mut_mat)], 2, function(x) as.numer
ic(as.character(x)))
 cancer_signatures_selected[,1:ncol(cancer_signatures_selected)] = apply(cancer_signa
tures_selected[,1:ncol(cancer_signatures_selected)], 2,
                                                        function(x) as.numeric(as.char
acter(x)))

 fit_res <- fit_to_signatures(mut_mat, as.matrix(cancer_signatures_selected))
 plot_contribution(fit_res$contribution,
                   cancer_signatures[,1:ncol(cancer_signatures)],
                   coord_flip = T,
                   mode = "absolute")
```

```
## Warning in contribution * total_signatures: longer object length is not a
## multiple of shorter object length
```

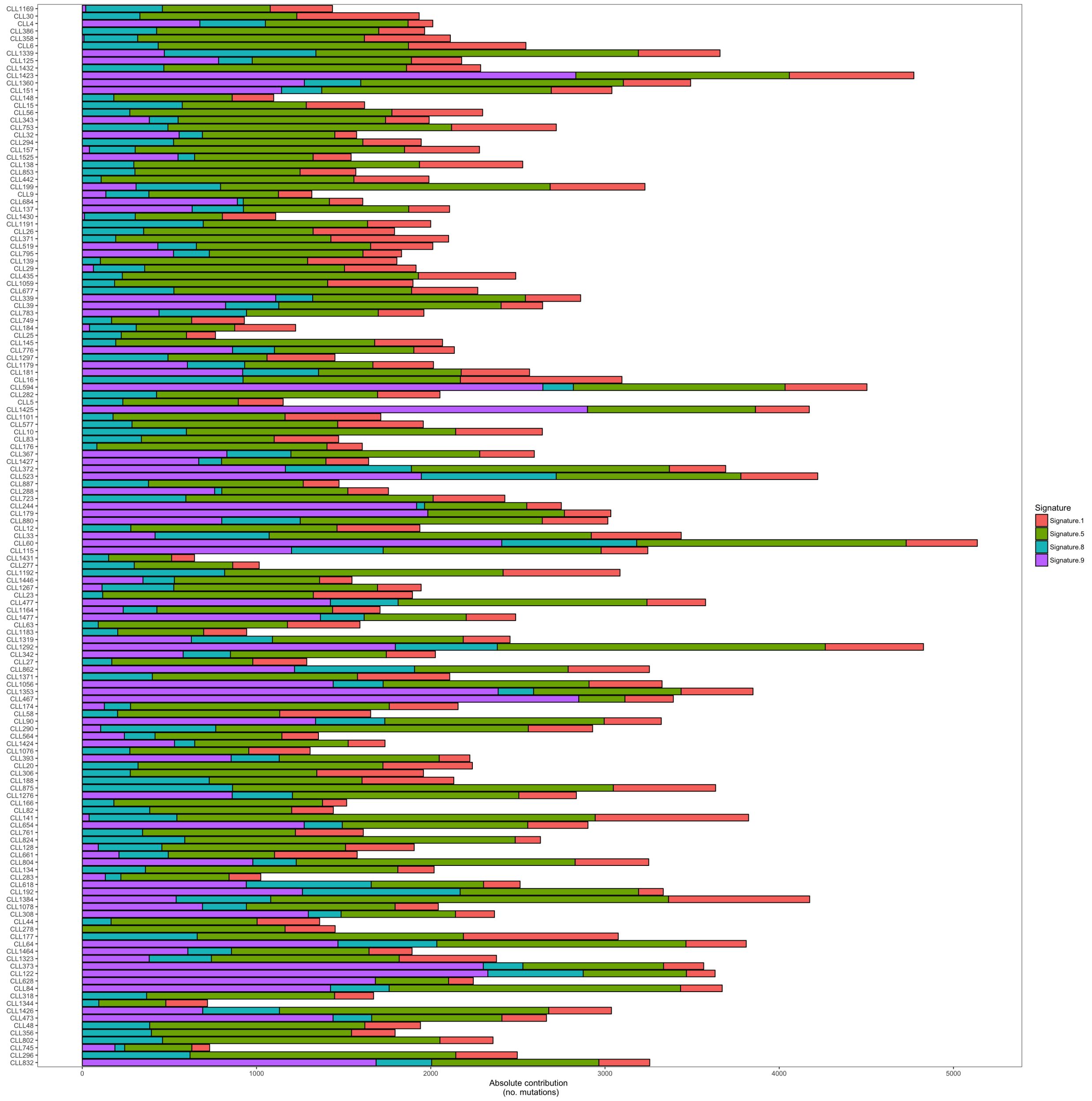

Note that the `echo = TRUE` parameter was added to the code chunk to prevent printing of the R code that generated the plot.
